# Submergence of the filamentous Zygnematophyceae *Mougeotia* induces differential gene expression patterns associated with core metabolism and photosynthesis

**DOI:** 10.1007/s00709-021-01730-1

**Published:** 2021-12-22

**Authors:** Janine M.R. Fürst-Jansen, Sophie de Vries, Maike Lorenz, Klaus von Schwartzenberg, John M. Archibald, Jan de Vries

**Affiliations:** 1 Department of Applied Bioinformatics, Institute for Microbiology and Genetics, University of Goettingen, Goldschmidtstr. 1, University of Goettingen, 37077 Goettingen, Germany; 2 Department of Experimental Phycology and SAG Culture Collection of Algae, Albrecht-von-Haller Institute for Plant Science, University of Goettingen, Nikolausberger Weg 18, 37073 Goettingen, Germany; 3 Institute of Plant Science and Microbiology, Microalgae and Zygnematophyceae Collection Hamburg (MZCH) and Aquatic Ecophysiology and Phycology, Universität Hamburg, Ohnhorststr. 18, 22609 Hamburg, Germany; 4 Department of Biochemistry and Molecular Biology, Dalhousie University, Sir Charles Tupper Medical Building, 5850 College Street, Halifax, NS B3H 4R2 Canada; 5 Goettingen Center for Molecular Biosciences (GZMB), University of Goettingen, Justus-von-Liebig-Weg 11, 37077 Goettingen, Germany; 6 Campus Institute Data Science (CIDAS), University of Goettingen, Goldschmidstr. 1, 37077 Goettingen, Germany

**Keywords:** Streptophyte algae, Charophytes, RNAseq, Algal culturing, Algal physiology

## Abstract

The streptophyte algal class Zygnematophyceae is the closest algal sister lineage to land plants. In nature, Zygnematophyceae can grow in both terrestrial and freshwater habitats and how they do this is an important unanswered question. Here, we studied what happens to the zygnematophyceaen alga *Mougeotia* sp., which usually occurs in permanent and temporary freshwater bodies, when it is shifted to liquid growth conditions after growth on a solid substrate. Using global differential gene expression profiling, we identified changes in the core metabolism of the organism interlinked with photosynthesis; the latter went hand in hand with measurable impact on the photophysiology as assessed via pulse amplitude modulation (PAM) fluorometry. Our data reveal a pronounced change in the overall physiology of the alga after submergence and pinpoint candidate genes that play a role. These results provide insight into the importance of photophysiological readjustment when filamentous Zygnematophyceae transition between terrestrial and aquatic habitats.

**Supplementary Information:**

The online version contains supplementary material available at 10.1007/s00709-021-01730-1.

## Introduction

Streptophyte algae diverged from the chlorophytes and prasinodermophytes between 700 and 1000 million years ago (Zimmer et al. 2007; Morris et al. 2018; Li et al. 2020). They form a paraphylum that is sister to the monophyletic Embryophyta, the land plants—together, land plants and streptophyte algae form the monophylum Streptophyta (Wickett et al. 2014). One of the most important questions in the field of land plant evolution is which particular lineage of streptophyte algae within this paraphylum represents the sister lineage to land plants. Streptophyte algae encompass a diverse range of organisms, including the unicellular Mesostigmatophyceae and Chlorokybophyceae (cell packages), consisting of only a few species (see also Irisarri et al. 2021), the unicellular and filamentous Klebsormidiophyceae (Mikhailyuk et al. 2015), and the streptophyte algae within Phragmoplastophyta that include morphologically complex multicellular organisms such as the Charophyceae—and the land plants. Various lines of evidence indicate that, among these Phragmoplastophyta, the Zygnematophyceae represent the sister lineage to land plants (Wodniok et al. 2011; Wickett et al. 2014; Leebens-Mack et al. 2019). It is thus of considerable interest what physiological properties these organisms possess—combined with data on land plants, such an understanding makes it possible to infer the physiology of the earliest land plants (Fürst-Jansen et al. 2020).

A key piece of the puzzle of understanding plant terrestrialization is the difference between growth in an aquatic environment and growth in a terrestrial habitat with limited water supply. Throughout the course of evolution, various algal lineages have mastered this so-called wet-to-dry transition. This is no small feat. The terrestrial habitat poses various challenges for a photosynthesizing organism, including fluctuations in abiotic factors such as temperature, water availability, and intensity and quality of irradiance (Foyer et al. 1994; Karsten et al. 2007; Holzinger et al. 2014; Ohama et al. 2017).

Terrestrial algae meet the challenges of their habitat with various physiological adaptations (Holzinger and Pichrtová 2016). These include the presence of mycosporine-like amino acids (MAAs) found in both chlorophyte and streptophyte algae. MAAs have UV-protecting properties. Among streptophyte algae, the Klebsormidiophyceae *Hormidiella* and *Klebsormidium* stand out by producing potent sunscreen MAAs with an absorption maximum at 325 and 324 nm (Kitzing and Karsten 2015). While such MAAs have not been reported for Zygnematophyceae, *Zygnema* spp. are known to produce phenolic compounds upon elevated UV irradiance (Pichrtová et al. 2013). Indeed, the unicellular Zygnematophyceae *Penium margaritaceum* was recently reported to contain flavonoids (Jiao et al. 2020). While the exact biochemical routes towards these metabolites are currently elusive, homologs of genes coding for core enzymatic biosynthetic steps that lead to relevant precursor metabolites in land plants (the phenylpropanoid pathway *sensu lato*) are also found in streptophyte algae (de Vries et al. 2017, 2021). Recently, Renault et al. (2019) highlighted the putative links between phenylpropanoid biosynthesis in streptophyte algae and shared ancestral chassis for producing hydrophobic polymers from which lignin, cutin, suberin, and sporopollenin arose. Indeed, Zygnematophyceae surround their zygotes with resistant polymers resembling sporopollenin (de Vries et al. 1983; Poulícková et al. 2007). Recently, Permann et al. (2021) employed glycan labeling as well as Raman spectroscopy to zygospores of *Mougeotia disjuncta* (which belongs to the same algal genus as the strains analyzed here); they found these zygospores to consist of a combination of carbohydrates, lipids, and aromatic compounds, speaking to sporopollenin-like material.

UV irradiance is not the only sunlight-associated challenge in the terrestrial habitat. Photosynthetically active radiance (PAR) reaches much higher levels on the surface of the earth as opposed to an aquatic environment, where the sunlight is buffered by the absorptive properties of water. One of the main mechanisms that mitigates damage to the components of the photosynthetic light reaction, in particular the vulnerable photosystem II, is non-photochemical quenching (NPQ; Müller et al. 2001; Jahns and Holzwarth 2012). The first and fastest response of NPQ is energy-dependent quenching (qE). Its activation hinges upon conformational changes in the photosystem and the detection of an altered pH in the thylakoid lumen (Krause et al. 1982). While their predominance varies across the green lineage, evidence suggests that the LHCSR (light-harvesting complex stress-related protein) and/or PSBS (photosystem II subunit S) proteins play a major role in this process (Li et al. 2000; Peers et al. 2009; Gerotto and Morosinotto 2013; Correa-Galvis et al. 2016). It is nevertheless prudent to note that some chlorophyte algae seem to lack qE (Christa et al. 2017). The result of NPQ is that superfluous energy, which cannot be meaningfully channeled into the light reaction chain, dissipates as harmless heat.

The role of NPQ and acclimation processes of the photosystem has been extensively studied in terrestrial streptophyte algae. For example, Herburger and Holzinger (2015) found that the photosynthetic effective quantum yield is strongly reduced in *Klebsormidium* strains upon desiccation but also recovers fully upon rehydration suggesting a high desiccation tolerance. Furthermore, Karsten et al. (2014) found that the sister group of *Klebsormidium*, *Interfilum*, also appears to have similar characteristics regarding high tolerance to stressors such as desiccation but also temperature that reflect in their photosynthetic physiology. That said, not only the family of Klebsormidiaceae shows this high tolerance to stressors. In the class of Zygnematophyceae, Holzinger et al. (2018) found that after UV-treatment in different *Zygnema* strains their effective quantum yield recovers completely in some cases. There are however other conserved mechanisms for photoprotection acting in algae. One is the expression of EARLY LIGHT INDUCED PROTEIN (ELIP). ELIPs are chlorophyll *a*/*b*-binding proteins that accumulate under stress and have a photoprotective function (Montané et al. 1997; Hutin et al. 2003). Elevated expression of ELIP-coding genes under light and temperature stress has now been reported for the Zygnematophyceae *Zygnema* and *Mougeotia* (de Vries et al. 2018; Rippin et al. 2019; de Vries et al. 2020). As with the relevance of NPQ under water scarcity, *ELIP* expression is also induced in desiccated *Zygnema* (Rippin et al. 2017). Thus, while we know about physiological responses of Zygnematophyceae challenged with water scarcity, we know very little about the reverse process—which is of similar importance for organisms that thrive in temporary water bodies. Plant terrestrialization likely entailed a repetition of several wet-to-dry and dry-to-wet transitions; therefore, investigating both transitions is important. Furthermore, living on land means a steady change between wet and dry conditions (rain, fog, and dew). *Mougeotia* spp. live in a variety of freshwater habitats, many of them are temporary habitats such as ditches and small temporary ponds.

In this study, we have used a laboratory controlled environmental shift approach to emulate what happens to the filamentous zygnematophyceaen alga *Mougeotia* sp., which predominantly lives in freshwater habitats, shortly after being submerged. Our data highlight photosynthesis-associated physiological responses and the global gene expression patterns that bring them to bear.

## Material and methods

### Culturing and treatment

For the RNAseq experiments, *Mougeotia* sp. MZCH 240 (which we obtained from the Microalgae and Zygnematophyceae Collection, Hamburg, Germany, [von Schwartzenberg et al. 2013]) was cultured as described in de Vries et al. (2020)—algae were grown for 7 days on modified freshwater F/2 (Guillard 1975) with 1% agar at 22°C and 120 μmol quanta m^-2^ s^-1^ from an LED light source (12h/12h light/dark cycle) in 9 cm plates. For submergence, 10 mL of temperature-adjusted liquid freshwater F/2 (Guillard 1975) were added to each agar plate; for RNAseq, algae were directly transferred into Trizol (Thermo Fisher, Walthm, MA, USA) after 4 h of submergence.

For the photophysiological experiments, *Mougeotia scalaris* strain SAG 164.80 (of the Culture Collection of Algae, Göttingen, Germany; Friedl and Lorenz 2012) and *Mougeotia* sp. MZCH 240 were grown on (i) WHM medium (*M. scalaris* SAG 164.80; Nichols 1973) with 1% agar or in liquid WHM medium or (ii) modified freshwater F/2 (*Mougeotia* sp. MZCH 240) with 1% agar or in liquid F/2 medium at 22°C and 120 μmol quanta m^-2^ s^-1^ from an LED light source (12h/12h light/dark cycle). For submergence, 10 mL of temperature-adjusted liquid WHM (*M. scalaris* SAG 164.80) or liquid F/2 (*Mougeotia* sp. MZCH 240) were added to each plate and *F*_v_/*F*_m_ was measured after various incubation timepoints (2h, 4h, 6h, 8h, 24h; plus 1h and 3h for *Mougeotia scalaris* SAG 164.80). For morphological observations and micrographs, *M. scalaris* SAG 164.80 and *Mougeotia* sp*.* SAG 650-1 were used as additional comparative material and grown either in liquid or solid for 9 weeks on Desmidiacean Medium (MiEB12; medium 7 of Schlösser 1994). For *Mougeotia* sp. MZCH 240, microscope pictures were taken after the 24h timepoint under the growing conditions described above.

### RNA extraction and sequencing

RNA extraction and sequencing procedures were described in de Vries et al. (2020). In brief, we extracted RNA in six biological replicates from the control samples and in biological triplicates from the liquid-treated samples. For RNA extraction, algae were directly transferred into 1 mL of Trizol using a sterilized spatula (Thermo Fisher, Waltham, MA, USA); extraction procedures were carried out in accordance to the protocol provided by the manufacturer. Isolated RNA was treated with DNAse I (Thermo Fisher), quality assessed on a formamide agarose gel, quantified using a Nanodrop spectrometer (Thermo Fisher), and shipped to Genome Québec (Montreal, Canada) for sequencing. There, RNA was quality checked again, using a Bioanalyzer (Agilent Technologies Inc., Santa Clara, CA, USA). Libraries were constructed using the NEB mRNA stranded Library preparation kit (New England Biolabs, Beverly, MA, USA), on the Illumina NovaSeq6000 platform.

### RNAseq analyses: data processing, statistics, KEGG, and GOterm

Initial processing of the RNAseq data was described in de Vries et al. (2020). In brief, reads were checked for quality using FASTQC version 0.11.7 (FASTQC 2018), trimmed with TRIMMOMATIC v0.36 (Bolger et al. 2014; settings: ILLUMINACLIP:TruSeq3-PE- 2.fa:2:30:10:2:TRUE HEADCROP:10 TRAILING:3 SLIDINGWINDOW:4:20 MINLEN:36), and quality checked again using FASTQC v0.11.7. For details on read data, see the “Data availability” section. The transcriptome assembly using the TRINITY pipeline (Haas et al. 2013), RSEM (RNA-Seq by Expectation Maximization)-based read mapping (Li and Dewey 2011) was carried out and described in de Vries et al. (2020).

Negative binomial distribution-based statistical analyses of the read counts were performed using edgeR version 3.28.0 (Robinson et al. 2010), taking the biological triplicates into account. For all downstream analyses, only gene expression changes with a Benjamini-Hochberg-corrected *p* value ≤ 0.001 and significantly elevated differential gene expression (log_2_ (fold change) ≤ −1 or log_2_ (fold change) ≥ 1) were considered.

For gene expression analysis based on KEGG orthologs, we worked with expression levels in TPM that were normalized via TMM (trimmed mean of *M* values; Robinson and Oshlack 2010). These data against KEGG pathways occur in land plants. If multiple transcripts had the same KEGG ortholog as their best hit, their expression values were combined—for the final calculations, a given KEGG ortholog had one TMM-normalized TPM value.

For GO term enrichment using GOrilla (Eden et al. 2009), we used AGI numbers obtained by querying the predicted *Mougeotia* proteins against Arabidopsis in a BLASTp in a comparison of two unranked list of genes. For this, we used all obtained Arabidopsis homologs (i.e. the best BLASTp hits) as the background set (as the whole transcriptome) and all significantly regulated genes as target set—one target set for all up-regulated genes, one for all downregulated genes.

### Photophysiology

All measurements of the maximum-quantum yield (*F*_v_/*F*_m_) were done using the maxi version of the Imaging-PAM (ImagMAX/L, M-series, Walz) with an IMAG-K5 CCD camera controlled with the ImagingWinGigE (V2.32) software. Treated as well as control samples were dark adapted 10–30 min before measurement. For *F*_v_/*F*_m_ measurement, a short saturation pulse with intensity 10 (setup 1-3; level 3 for setup 4) was applied, which is the standard intensity for the IMAGING-PAM. Within the four experimental setups (three with SAG 164.80 and a fourth with MZCH 240), the settings for measuring light and gain were adjusted slightly (setup 1: measuring light 4, gain 2; setup 2: measuring light 1, gain 10; setup 3(+setup 4): measuring light 1, gain 3). A special SP-Routine was not applied to modify the signal to noise ratio of the fluorescence measurement. Statistical analysis was done using Mann-Whitney *U* tests (Mann and Whitney 1947) with R (version 3.6.1).

### Phylogenetic analysis

To explore whether the ABA3 and PAP homolog we detected in the RNA-Seq-based de novo assembly represents an ABA3 ortholog in *Mougeotia* sp. MZCH 240, we used BLASTp to mine the protein datasets of (i) the land plants *Anthoceros agrestis* (Li et al. 2020), *Arabidopsis thaliana* (Lamesch et al. 2012), *Azolla filiculoides* (Li et al. 2018), *Marchantia polymorpha* (Bowman et al. 2017), *Nicotiana tabacum* (Sierro et al. 2014), *Physcomitrium patens* (Lang et al. 2018), *Selaginella moellendorffii* (Banks et al. 2011); (ii) the streptophyte algae *Chlorokybus atmophyticus* CCAC 0220 (Wang et al. 2020), *Chara braunii* S 276 and S 277 (Nishiyama et al. 2018), *Klebsormidium nitens* NIES-2285 (Hori et al. 2014), *Mesotaenium endlicherianum* SAG 12.97 (Cheng et al. 2019), *Mesostigma viride* CCAC 1140 (Wang et al. 2020), *Spirogloea muscicola* CCAC 0214 (Cheng et al. 2019); (iii) *Bathycoccus prasinos* RCC 1105 (Moreau et al. 2012)*, Chlamydomonas reinhardtii* CC-503 (Merchant et al. 2007), *Volvox carteri f. nagariensis,* Eve (Prochnik et al. 2010).

All obtained sequences were aligned using MAFFT (Katoh and Standley 2013) with the L-INS-I settings. The alignment was used for computing a maximum likelihood phylogeny using IQ-TREE multicore v.1.5.5 for Linux 64-bit built (Nguyen et al. 2015) with 100 bootstrap replicates; the best model for protein evolution (WAG+F+I+G4 for ABA3 and WAG+I+G4 for PAP; both were chosen according to Bayesian Information Criterion) was determined using ModelFinder (Kalyaanamoorthy et al. 2017).

## Results and discussion

### Submergence in liquid medium triggers the differential expression of core metabolism and photosynthesis-related genes in *Mougeotia* sp.

Using the filamentous zygnematophycean alga *Mougeotia* sp. (a representative species of the zygnematophycean clade), we analyzed differences in the transcriptome of *Mougeotia* sp. MZCH 240 under two growth conditions: (i) growth on solid medium and (ii) 4 h after submergence with liquid medium.

**Fig. 1:**
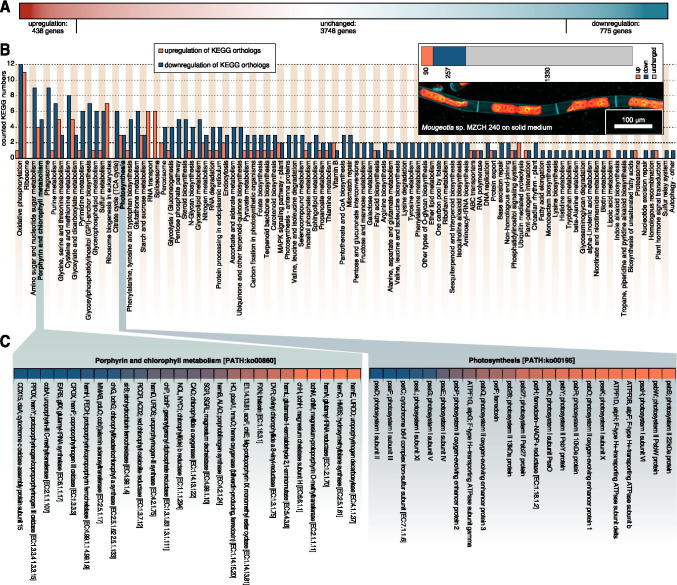
Global gene expression patterns in *Mougeotia* sp. MZCH 240. **A** Gradient-colored depiction (red up-regulated, white unchanged, and blue downregulated genes) of the differential global gene expression profile of all 4961 genes analysed in this study; the differential responses were obtained by comparing global gene expression of *Mougeotia* sp. MZCH 240 cultured on solid medium and submerged for 4h versus control (growth on solid medium). **B** Gene expression pattern of various KEGG orthologs in *Mougeotia* sp. MZCH 240. Biological replicates (at least triplicates) of gene expression data (TPM_TMM-normalized_) were summed up and set relative to the control condition data (submergence/control) and then mapped against the Kyoto Encyclopedia of Genes and Genomes (KEGG). An up- or downregulation of a KEGG ortholog was considered if it had a ≥ 2-fold change in gene expression levels. A bar diagram depicts the numbers of all up- (orange) or downregulated (dark blue) KEGG orthologs in the 118 detected KEGG plant pathways in *Mougeotia* sp. MZCH 240 4h after being submerged (shift) in liquid medium compared to the control culture, which was kept on solid medium. On the upper right side all counted KEGG numbers from up- (90) or down- (257) regulated KEGG orthologs are shown in a stacked bar plot together with 1330 KEGG orthologs with unchanged (grey) gene expression patterns; below is a confocal micrograph of *Mougeotia* sp. MZCH 240 under control conditions (grown in modified freshwater F/2 with 1% agar 22°C and 120 μmol quanta m-2 s-1)—cell walls were made visible using 1% calcofluor white staining (teal false colored), the plastids are shown in a false-colored red-orange gradient based on their chlorophyll *a* autofluorescence. **C** A heatmap of the gene expression patterns in *Mougeotia* sp. MZCH 240 of the two KEGG plant pathways “Porphyrin and chlorophyll metabolism [PATH:ko00860]” and “Photosynthesis [PATH:ko00195]” in detail. Data is shown as log_2_ (fold change_submergence/control_) in a color gradient ranging from dark blue (downregulation) to orange (upregulation). Unchanged expression levels are not depicted here

Using the Illumina NovaSeq 6000 platform (operated by Genome Quebec), we obtained ~159 million paired reads for the solid growth condition (6 biological replicates) and 100 million paired reads for the sample taken 4h after submergence (3 biological replicates). After quality checking and trimming, we mapped these reads onto the transcriptome assembly of *Mougeotia* sp. MZCH 240 (de Vries et al. 2020) using the RSEM toolkit included in the TRINITY pipeline. Using this transcriptome assembly, we worked with 4961 genes, of which 438 genes showed more than 2-fold upregulation and 775 genes showed more than 2-fold downregulation (Figure 1A; more on statistic scrutinization below).

First, we were interested in getting an overview over transcriptomic differences induced by submergence in liquid medium; we used the pathway framework of the Kyoto Encyclopedia of Genes and Genomes (KEGG) database. We used BLASTKOALA (Kanehisa et al. 2016) to identify KEGG orthologs among our de novo assembled transcripts and then linked the expression values (fold change) to the corresponding KEGG numbers. All gene expression values for a given KEGG ortholog were summed up as described in de Vries et al. (2020). A KEGG ortholog was considered up- or downregulated if it had a ≥ 2-fold change in gene expression level. 118 KEGG pathways were identified (Figure 1B). In total, expression values for 1677 KEGG orthologs (corresponding 1176 unique KEGG orthologs) were mapped across pathways, among which 90 orthologs were up-regulated and 257 downregulated in *Mougeotia* sp. MZCH 240 after the shift to liquid conditions; this adds up to a total of 347 responsive KEGG orthologs while 1330 orthologs showed an unchanged response (see the overview in the top right section of Figure 1B).

Most prominent among the top 20 most responsive KEGG pathways were those associated with core metabolic processes such as “oxidative phosphorylation [PATH:ko00190]”, “ribosome [PATH:ko03010]”, and “amino sugar and nucleotide sugar metabolism [PATH:ko00520]” with 13, 11, and 9 differentially regulated KEGG orthologs respectively. We interpret categories such as ribosome, nucleotide metabolism, and any amino acid metabolism as a readout often observed upon any treatment/shift in environmental conditions: the basal molecular machineries of the cells are responding: they power up for making a range of new/different proteins, resulting in a need to produce a different set of amino acids for making these; prior, as well as alongside of this, they make, process, and transport RNA. Similarly, the downregulation of respiration (oxidative phosphorylation and the citrate cycle) can likely be traced to an overall impacted metabolism. We hence searched whether the data speak to any such process upstream and honed in on photosynthesis—the source of carbon for any photoautotroph.

Two photosynthesis-related pathways, namely, “Porphyrin and chlorophyll metabolism [PATH:ko00860]” (4^th^ most responsive, when considering both up- and downregulated KEGG orthologs) and “Photosynthesis [PATH:ko00195]” (16^th^ most responsive), contained some of the most highly differentially regulated KEGG orthologs among all 118 pathways; with 4 up- and 5 downregulated KEGG orthologs for the Porphyrin and chlorophyll metabolism pathway and 3 up- and 3 downregulated KEGG orthologs for the photosynthesis pathway (Figure 1C). The finding of photosynthesis-associated genes might explain why other pathways of core metabolism, as well as housekeeping genes are also affected—photosynthesis is at the heart of plant and algal physiology. If the primary fixation of carbon mediated by photosynthesis is affected by a changing environment, it is conceivable that other pathways dependent on the fixed carbon tag along.

### Submergence in liquid medium impacts the photophysiology of two strains of *Mougeotia*

**Fig. 2:**
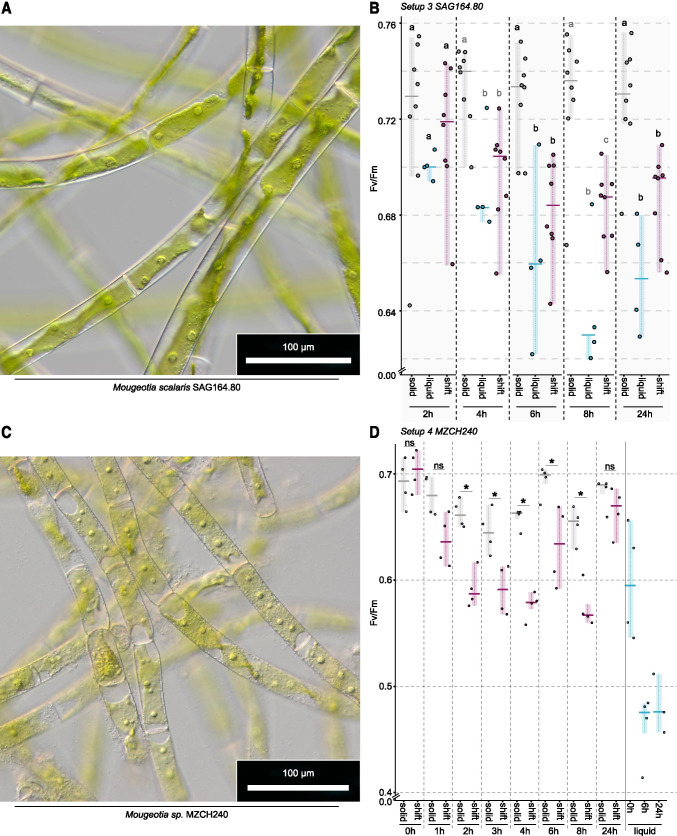
Plastid morphology and photophysiological characteristics (*F*_v_/*F*_m_) in *Mougeotia scalaris* SAG 164.80 and *Mougeotia* sp. MZCH 240. **A** Light micrograph of *M. scalaris* SAG 164.80 in liquid medium. **B** Maximum PSII quantum yield (*F*_v_/*F*_m_) in *M. scalaris* SAG 164.80 solid- and liquid-medium control samples (grown for 7 days on WHM-Medium at 20°C, 120 μmol quanta m^-2^ s^-1^) as well as samples treated with the liquid shift—which were grown on solid medium and submerged in 10 ml liquid medium. **C** Light micrograph of *Mougeotia* sp. MZCH 240 24h after submergence. **D** *F*_v_/*F*_m_ values for *Mougeotia* sp. MZCH 240 when grown on F/2 medium for 7days at 22°C, 120μmol quanta m^-2^ s^-1^ on solid and liquid medium. Liquid shift was achieved by adding 10 ml liquid medium to algal cultures grown on solid medium. *F*_v_/*F*_m_ values were collected at 0, 1, 2, 3, 4, 6, 8, and 24 h after the shift and for the control on solid medium. Owing to the low growth rate in liquid medium values for *F*_v_/*F*_m_ were measured only at 0, 6, and 24 h for liquid cultures of *Mougeotia* sp. MZCH 240. *F*_v_/*F*_m_ for **B** and **D** was measured from the same sample at several time points (from 2h up to 24h) after liquid medium was added by using an ImagMAX/L PAM with an IMAG-K5 CCD camera (for details, see the “Material and methods” section). Solid control samples are depicted in grey, liquid control samples are shown in blue, and liquid-treated samples (shift) are depicted in pink. Statistical analysis was done using Mann-Whitney *U* tests with R (version 3.6.1); significant differences at *p* < 0.05 are depicted using letters and asterisks

The top three up- and downregulated KEGG orthologs that belong to the pathway “photosynthesis” mainly fall into the category of photosystem I and II subunits, which suggests pronounced readjustment of the composition and stoichiometry of main components that form the chain of proteins acting in the photosynthetic light reaction; this likely goes hand in hand with selectively elevated turnover rates. We thus honed in on the plastid-associated biology of *Mougeotia*. For this, we made use of the emerging model system *M. scalaris* SAG 164.80 (Regensdorff et al. 2018; Figure 2A) and investigated its photophysiological changes after submergence using PAM. For this, we used three experimental setups, each with a minimum of three replicates. In a first experimental setup, we tested changes in maximum quantum yield (*F*_v_/*F*_m_) over time when *M. scalaris*. was grown on plates and in liquid culture. We initially explored whether photophysiological changes occur over a short period (4h, Figure S1A; setup 1) during daily growth; in the second setup, we investigated whether there are differences in daily performance (24h, Figure S1B; setup 2). On solid medium, *F*_v_/*F*_m_ appeared stable when measurements were only 4h apart, yet when tested daily, we found a decrease in *F*_v_/*F*_m_ in the algal culture (*p* value= 0.029; Figure S1A and B). In liquid culture, *F*_v_/*F*_m_ increased from 0.382±0.020 to 0.412±0.018 after 4h (*p* value =0.041) in setup 1 but was similar to the starting value after 24h in setup 2 (0.613±0.017 to 0.632±0.015, *p* value = 0.0343; Figure S1A and B). We, however, noted that *F*_v_/*F*_m_ of *M. scalaris* SAG 164.80 differed significantly at the first measurement (solid 0h: *p* value =0.029; liquid 0h: *p* value =0.0095).

Despite differences in the actual values of *F*_v_/*F*_m_ in the algal culture, we observed a similar trend after submergence of the algae on plate. Short after submergence (1h), *F*_v_/*F*_m_ was similar to that of algal culture grown on non-submerged plates. That said, over time, we saw a decrease of *F*_v_/*F*_m_ that significantly differs from that of algae grown on agar after 4h (Figure S1A and B). It is noteworthy, however, that the values between liquid culture, solid culture and the submerged culture are similar at 24h (Figure S1B). The data thus remained inconclusive because only two time points were sampled for liquid- and solid-grown algae and the time points were taken from different cultures.

In a next step, we (i) traced the photophysiological properties of the same liquid-grown, solid-grown and submerged algal cultures over time and (ii) compared the differences in *F*_v_/*F*_m_ between the different growth conditions (setup 3; Figure 2B) at a given time point. Both solid and liquid grown cultures remained steady over time in their *F*_v_/*F*_m_ (Table 1). In contrast, the submerged cultures tend to have a significantly decreased *F*_v_/*F*_m_ after 6, 8, and 24h compared to the *F*_v_/*F*_m_ at 2h. This agrees with the decreasing trend observed for *F*_v_/*F*_m_ in the first two experiments, where different cultures were measured at the different time points. Additionally, this shows that while the decrease in *F*_v_/*F*_m_ for the submergence was real, the differences between the different time points for cultures grown in liquid or on solid medium stems from fluctuations in cultures and culturing.

**Table 1:** Statistical analysis of maximum quantum yield in *M. scalaris* SAG 164.80 over time. Numbers denote *p* values obtained through Mann-Whitney *U* tests

	*Solid 2h*	*Solid 4h*	*Solid 6h*	*Solid 8h*
*Solid 4h*	0.1508			
*Solid 6h*	0.4406	0.7789		
*Solid 8h*	0.1484	0.726	0.7344	
*Solid 24h*	0.3828	0.1953	0.5469	0.8332
	*Liquid 2h*	*Liquid 4h*	*Liquid 6h*	*Liquid 8h*
*Liquid 4h*	0.875			
*Liquid 6h*	0.25	0.125		
*Liquid 8h*	0.09751	0.125	0.125	
*Liquid 24h*	0.125	0.125	0.625	0.25
	*Shift 2h*	*Shift 4h*	*Shift 6h*	*Shift 8h*
*Shift 4h*	0.05469			
*Shift 6h*	**0.01563**	0.07813		
*Shift 8h*	**0.02917**	0.07593	0.833	
*Shift 24h*	**0.02071**	**0.03906**	0.3615	0.5541

We next compared the data from a given time point between the different growth conditions. While *F*_v_/*F*_m_ did not differ at 2h, it was always higher in solid grown medium than in liquid and submerged cultures from 4h onwards (Figure 2B). Liquid and submerged cultures showed mainly similar *F*_v_/*F*_m_ values, the only exception being 8h after treatment; at this time point, the liquid cultures had a significantly lower *F*_v_/*F*_m_ than the submerged culture. Taken together, our data suggest that submerged cultures behave—after an initial equilibration phase—more similar to cultures grown in liquid medium than on solid medium. While the trend is largely reproducible, cultural fluctuations in initial photosystem performance nevertheless exist.

In order to scrutinize whether the observations we made on *Mougeotia scalaris* SAG 164.80 (Figure 2A and B) also hold for the strain on which the transcriptomic analyses were performed, we carried out the PAM-based investigations with *Mougeotia* sp. MZCH 240. The cultures of MZCH 240 had *F*_v_/*F*_m_ values at the start of the experiment that were (a)similar for the cultures (grown on solid 1% agar medium) that were about to be submerged (shift) and those that were kept as the untreated control (solid) (0.703±0.017 (shift) and 0.691±0.020 (solid), no significant difference) and (b) comparable to the values of the strain SAG164.80. Cultures of MZCH 240 grown in liquid medium generally had lower *F*_v_/*F*_m_ values [0.598±0.053 (*t*_0_), 0.462±0.033(6h), and 0.482±0.028(24h)]. Already after 2h, submerged cultures had significantly (*p*=0.029) lower *F*_v_/*F*_m_ values; this trend of significantly lower (*p*<0.05) *F*_v_/*F*_m_ values continued at time points 3h, 4h, 6h, and 8h. After 24h, the submerged cultures appeared to have acclimated to their new culturing conditions as the *F*_v_/*F*_m_ values were almost back to *t*_0_: 0.665±0.019 (shift) and 0.682±0.013 (solid)—with no significant difference. This is in contrast to the physiological behavior of SAG164.80, which did not acclimate to submergence within a 24h timeframe. Regardless, it should be re-iterated that MZCH 240 showed significantly lower *F*_v_/*F*_m_ values at 4h after submergence, which is the time point that was used for transcriptome analyses of this strain; both MZCH 240 and SAG164.80 behaved alike at this time point with regard to their photophysiology assessed through *F*_v_/*F*_m_.

**Fig. 3:**
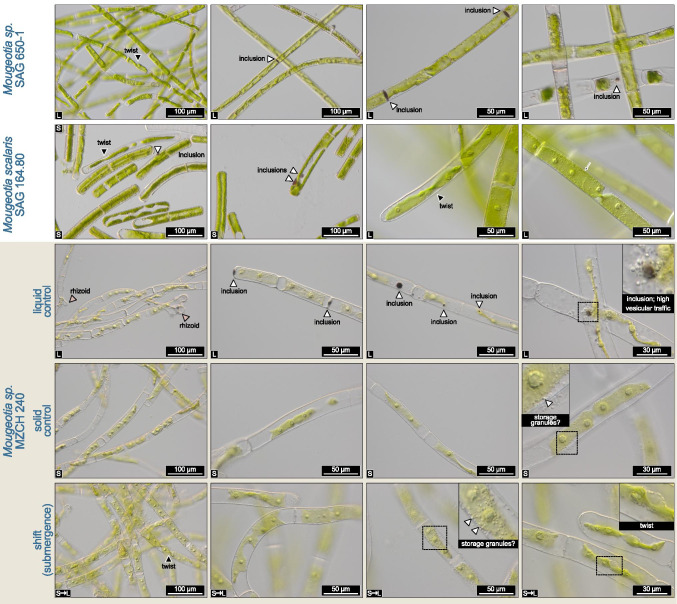
Notable observations in three *Mougeotia* strains. Nomarski interference contrast micrographs of the strains *Mougeotia* sp. SAG 650-1, *Mougeotia scalaris* SAG 164.80, and *Mougeotia* sp. MZCH 240; the latter was grown in liquid medium, on solid agar plates, and on agar plates and subjected to 24h of submergence in liquid medium (“shift”). The two SAG strains 650-1 and 164.80 were grown either in liquid or on solid MiEB12 Medium, as indicated by the “L” (liquid medium) or “S” (solid medium) on the bottom left side of the pictures. Notable phenotypic observations include: (a) darkly colored inclusions (sometimes co-occurring with high density of intracellular bodies being trafficked); (b) rhizoid formation in liquid culture; (c) formation of granules, possibly for storage. Also note the twisting chloroplasts, including “edge-on” orientations as a sign for functional chloroplast movement induced by microscope illumination. Labels in the bottom left corner denote: L=liquid-grown, S=solid-grown (agar), S➞L=solid-grown and submerged for 24h

While the photophysiology had recovered at 24h after submergence, only then did morphological differences between the solid control and submerged cultures emerge in *Mougeotia* sp. MZCH 240. The shifted cultures more readily accumulated storage granules (Figure 3); whether these might speak to lipid droplets, as potentially occurring in *Spirogyra* (see also de Vries and Ischebeck 2020), is unclear. Such granules were sometimes also found in samples of the solid control group. However, the most notable phenotypes were visible in the liquid-grown cultures. Here, we observed rhizoid formation as well as brownish inclusions. Indeed, such inclusions also appeared in solid-grown SAG164.80 as well as liquid-grown SAG 650-1—the latter of which is a strain relative of MZCH 240. Despite them being strain relatives, we noticed that the strain MZCH 240 appeared to have a lighter chlorophyllous hue than SAG 650-1, which is however consistent with our previous experience in culturing MZCH 240 (see de Vries et al. 2020).

Together with the gene expression responses, the photophysiological data highlight the fact that the photosynthetic machinery of *Mougeotia* responds to the submergence of the algal filaments in liquid medium. We hence next explored which specific genes might be the key players among these changes.

### Responsiveness of genes for light-harvesting components, pigment biosynthesis, and starch metabolism following submergence of *Mougeotia* sp. MZCH 240

**Fig. 4:**
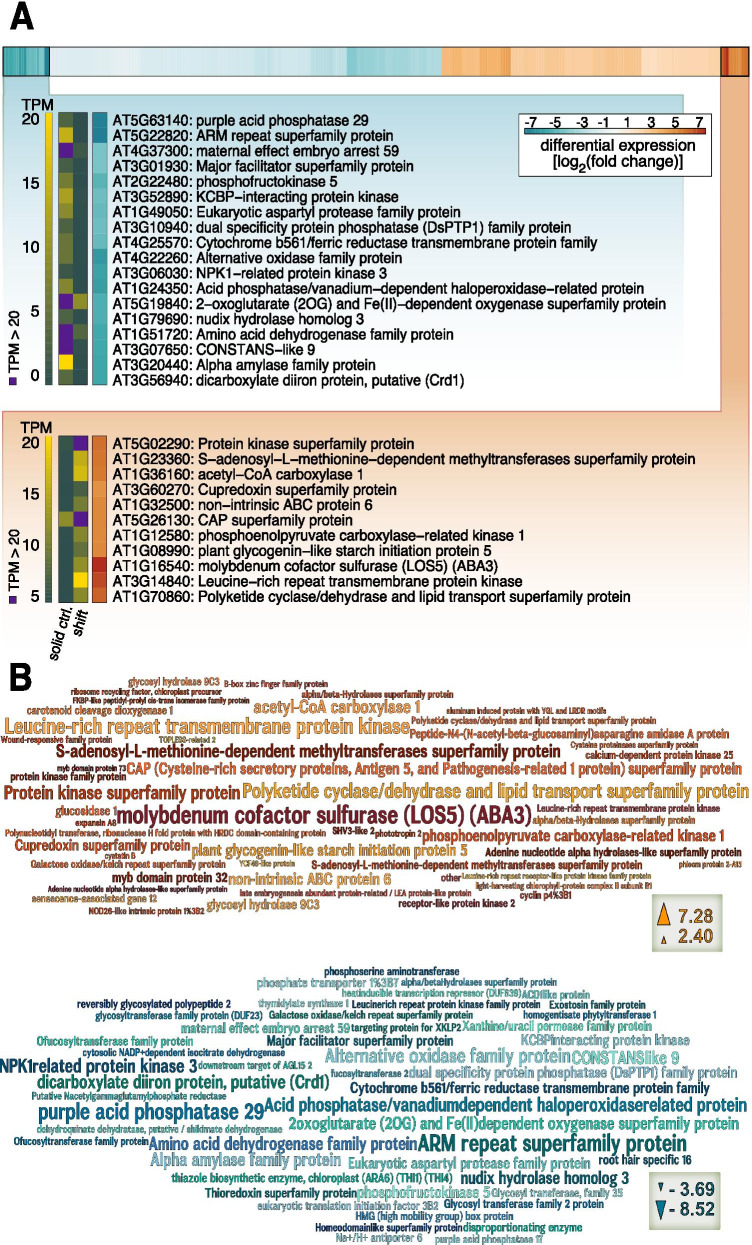
Top up-/downregulated genes in *Mougeotia* sp. MZCH 240 cultured on solid medium and submerged for 4h versus control on solid medium. **A** A heatmap with all up- (red) or downregulated (blue) genes in *Mougeotia* sp. MZCH 240 based on edgeR analysis of the RNAseq data. Only genes with a significant (Benjamini-Hochberg corrected *p* < 0.001) differential change in gene expression of 2-fold (all differential data are shown as log_2_[fold change _submergence/control_], calculated using edgeR) were considered. Using the R package *pheatmap* the data were sorted and log_2_ values of clusters of genes with the highest/lowest differential gene expression values are shown. The names and descriptions of corresponding *Arabidopsis thaliana* gene orthologs [prediction based on the reciprocal best BLAST hit (RBBH)] are displayed as well as the corresponding TPM (Transcript per million) values which are shown in a different color gradient (green to yellow). TPM values > 20 are colored in purple; shift = submergence, ctrl. = control. **B** Word clouds of the top 50 up- (red and orange colors) and top 50 downregulated (blue colors) genes in *Mougeotia* sp. MZCH 240 generated with Wordle and based on log_2_(fold change _submergence/control_), calculated with edgeR. The words represent the names and/or description of Arabidopsis orthologs (prediction based on the RBBH) and the word size corresponds to the differential gene expression change

To understand which gene expression changes were most pronounced upon submergence, we made use of homology searches against the well-annotated genome of *Arabidopsis thaliana* in combination with the differential transcript abundance elicited by submergence of *Mougeotia* sp. MZCH 240. For differential gene expression analyses, we considered only genes that had a Benjamini-Hochberg corrected *p* < 0.001 and a differential gene expression change of at least 2-fold (Figure 4). Overall, using these criteria, submergence triggered the upregulation of 120 genes (Table 2) and the downregulation of 171 genes (Supplementary Figure S2). Again, photosynthesis-related gene expression patterns stood out—both concerning genes relevant to the light reaction and those of downstream processes, such as three genes putatively coding for chlorophyll a/b-binding proteins (4.3-fold, 4.0-fold, and 3.9-fold upregulation) and a gene putatively encoding a light-harvesting component showed induction (Figure S1; 5.8-fold upregulation).

**Table 2:** 120 transcripts that significantly increased in abundance upon submergence in *Mougeotia* sp. MZCH 240

Mousp ID	Best *A.t. hit*	Annotation	log_2_(FC)	FDR
Mousp14158_c0_g1_i8	AT1G16540	molybdenum cofactor sulfurase (LOS5) (ABA3)	7.27520966	8.0067E-24
Mousp17078_c0_g3_i2	AT3G14840	Leucine-rich repeat transmembrane protein kinase	6.50770827	3.1038E-22
Mousp17366_c0_g1_i1	AT1G70860	polyketide cyclase/dehydrase and lipid transport	5.97865925	1.8507E-17
Mousp12113_c0_g1_i4	AT5G02290	protein kinase superfamily protein	5.60368112	9.185E-29
Mousp17366_c0_g3_i1	AT1G23360	S-adenosyl-l-methionine-dependent methyltransferases	5.40238661	9.7774E-09
Mousp17745_c1_g1_i11	AT1G36160	acetyl-CoA carboxylase 1	5.37604779	4.3006E-25
Mousp17215_c0_g5_i2	AT1G32500	non-intrinsic ABC protein 6	4.89640786	3.5159E-10
Mousp13170_c0_g1_i1	AT1G12580	phosphoenolpyruvate carboxylase-related kinase 1	4.84629602	8.928E-17
Mousp17366_c0_g2_i3	AT1G08990	plant glycogenin-like starch initiation protein 5	4.80921473	1.6156E-11
Mousp15175_c0_g2_i6	AT5G26130	Cysteine-rich secretory, Antigen 5, and Pathogenesis-related 1	4.76084995	9.7774E-09
Mousp14442_c0_g1_i1	AT3G60270	Cupredoxin superfamily protein	4.52531814	1.3694E-09
Mousp15384_c0_g2_i2	AT4G34990	myb domain protein 32	4.09225758	4.8567E-33
Mousp17772_c0_g1_i14	AT4G11050	glycosyl hydrolase 9C3	3.99810327	2.8402E-10
Mousp16800_c0_g1_i6	AT1G67490	glucosidase 1	3.81940181	1.062E-13
Mousp17501_c0_g1_i5	AT3G14920	Peptide-N4-(*N*-acetyl-beta-glucosaminyl) asparagine amidase A	3.7028685	1.4233E-09
Mousp17241_c0_g1_i6	AT1G16650	S-adenosyl-l-methionine-dependent methyltransferases	3.54376221	2.6424E-07
Mousp16885_c0_g1_i12	AT3G58450	Adenine nucleotide alpha hydrolases-like	3.43211036	3.7452E-12
Mousp14398_c0_g1_i5	AT3G63520	carotenoid cleavage dioxygenase 1	3.40138953	0.00008287
Mousp17219_c4_g1_i2	AT5G26150	protein kinase family protein	3.27932949	2.8364E-10
Mousp12560_c0_g1_i2	AT2G35890	calcium-dependent protein kinase 25	3.2585986	1.3769E-06
Mousp13723_c0_g1_i1	AT5G45890	senescence-associated gene 12	3.24606174	2.5541E-09
Mousp17422_c0_g1_i1	AT4G11050	glycosyl hydrolase 9C3	3.22808696	0.000010803
Mousp17708_c0_g2_i11	AT3G02130	receptor-like protein kinase 2	3.18914671	0.000023637
Mousp15137_c0_g1_i4	AT2G42450	alpha/beta-Hydrolases superfamily protein	3.10782404	4.3225E-13
Mousp13988_c0_g1_i5	none	none	3.10330699	6.974E-15
Mousp16061_c0_g3_i2	AT1G16250	Galactose oxidase/kelch repeat	3.06024435	1.1042E-16
Mousp16214_c0_g1_i1	AT2G44740	cyclin	3.05199502	2.5095E-15
Mousp14673_c1_g1_i14	AT1G66970	SHV3-like 2	2.98725544	2.3537E-07
Mousp17516_c0_g3_i2	AT4G20140	Leucine-rich repeat transmembrane protein kinase	2.93958975	2.9611E-09
Mousp17666_c0_g1_i1	AT5G20520	alpha/beta-Hydrolases superfamily protein	2.89768053	1.0882E-06
Mousp16146_c1_g1_i34	AT1G55960	Polyketide cyclase/dehydrase and lipid transport	2.89145041	5.3538E-08
Mousp15641_c0_g6_i2	AT2G32415	Polynucleotidyl transferase, ribonuclease H with HRDC domain	2.78988514	9.3814E-07
Mousp15363_c0_g1_i15	AT5G58140	phototropin 2	2.75782905	2.2375E-07
Mousp16839_c0_g1_i4	AT1G19660	Wound-responsive family protein	2.75208096	0.000021332
Mousp16811_c0_g3_i2	AT2G40610	expansin A8	2.73396604	0.00082134
Mousp13223_c0_g1_i6	AT4G18910	NOD26-like intrinsic protein 1%3B2	2.7251728	1.2886E-10
Mousp15049_c1_g5_i1	AT2G21320	B-box zinc finger family protein	2.68212139	7.8998E-14
Mousp15748_c2_g2_i3	AT3G19430	late embryogenesis abundant	2.66385694	3.9849E-08
Mousp16895_c0_g1_i9	AT3G19400	Cysteine proteinases superfamily protein	2.52775348	0.00051691
Mousp17457_c0_g2_i6	AT3G63190	ribosome recycling factor, chloroplast precursor	2.52623134	3.4766E-12
Mousp16770_c0_g2_i2	AT2G34430	light-harvesting chlorophyll-protein complex II subunit B1	2.52608987	0.00040624
Mousp17814_c0_g1_i1	AT3G12490	cystatin B	2.51128417	3.9795E-09
Mousp15345_c0_g4_i4	AT3G22850	aluminum induced protein with YGL and LRDR motifs	2.49160672	8.5789E-07
Mousp16831_c0_g1_i5	AT4G08850	Leucine-rich repeat receptor-like protein kinase	2.47066699	2.3962E-07
Mousp12430_c0_g1_i4	AT4G37260	myb domain protein 73	2.44626078	2.1144E-07
Mousp17048_c2_g5_i2	AT4G22830	YCF49-like protein	2.43736515	1.4633E-09
Mousp13966_c0_g1_i1	AT3G16830	TOPLESS-related 2	2.42899009	2.509E-07
Mousp12564_c0_g1_i1	AT3G53000	phloem protein 2-A15	2.4230952	3.8864E-10
Mousp15097_c0_g1_i4	AT2G43560	FKBP-like peptidyl-prolyl cis-trans isomerase	2.42256699	1.2101E-10
Mousp15383_c0_g1_i2	AT1G09740	Adenine nucleotide alpha hydrolases-like	2.39874357	0.00028434
Mousp17870_c0_g2_i4	AT2G46580	Pyridoxamine 5′-phosphate oxidase	2.39561141	1.7197E-12
Mousp15748_c2_g3_i4	AT3G19430	late embryogenesis abundant	2.39052414	5.2306E-08
Mousp17536_c0_g2_i15	AT4G16760	acyl-CoA oxidase 1	2.37665903	0.000010184
Mousp17563_c0_g2_i4	AT1G13980	sec7 domain-containing protein	2.36154393	0.00012739
Mousp17005_c2_g1_i2	AT5G54370	late embryogenesis abundant	2.34756478	0.000050599
Mousp17009_c0_g2_i11	AT4G33010	glycine decarboxylase P-protein 1	2.34716303	0.000027572
Mousp13949_c0_g1_i1	AT5G19360	calcium-dependent protein kinase 34	2.3443775	0.00023592
Mousp14435_c0_g1_i1	AT3G22750	Protein kinase superfamily protein	2.29061994	1.2152E-11
Mousp16006_c1_g2_i6	AT2G15010	Plant thionin	2.28071231	0.000001202
Mousp17583_c1_g1_i5	AT1G08550	non-photochemical quenching 1	2.26244193	6.0714E-06
Mousp17901_c2_g2_i8	AT5G14580	polyribonucleotide nucleotidyltransferase	2.24801086	0.00008991
Mousp15753_c0_g1_i21	AT2G34260	transducin family protein / WD-40 repeat	2.24643147	7.4559E-07
Mousp15748_c2_g4_i7	AT3G19430	late embryogenesis abundant	2.24637151	6.5626E-06
Mousp15175_c0_g1_i6	AT2G14610	pathogenesis-related protein 1	2.24594398	0.000030745
Mousp17901_c2_g3_i1	none	none	2.24550218	0.000023275
Mousp16876_c0_g5_i2	AT3G52140	tetratricopeptide repeat (TPR)-containing protein	2.22759061	0.000030632
Mousp17754_c1_g2_i1	AT5G41460	transferring glycosyl group transferase (DUF604)	2.22550195	1.2101E-10
Mousp10496_c0_g1_i1	AT4G33880	ROOT HAIR DEFECTIVE 6-LIKE 2	2.21640294	7.7321E-10
Mousp14422_c0_g1_i6	AT1G14870	PLANT CADMIUM RESISTANCE 2	2.21344381	8.4799E-06
Mousp13841_c0_g1_i3	AT2G24440	selenium binding protein	2.1868762	1.7433E-10
Mousp17685_c0_g1_i2	AT4G00260	Transcriptional factor B3 family protein	2.173197	0.00032902
Mousp17103_c0_g2_i3	AT2G37560	origin recognition complex second largest subunit 2	2.15944614	0.000088228
Mousp14784_c2_g1_i6	AT2G21940	shikimate kinase 1	2.14189653	4.7278E-09
Mousp16295_c0_g1_i5	AT1G31420	Leucine-rich repeat protein kinase	2.14136017	0.00020268
Mousp17228_c0_g3_i14	AT2G25185	Defensin-like (DEFL) family protein	2.13194267	0.00075013
Mousp14776_c0_g1_i2	AT5G15330	SPX domain-containing protein 4	2.12256734	3.8238E-10
Mousp15459_c0_g2_i1	AT1G44575	Chlorophyll A-B binding family protein	2.11945161	1.1268E-07
Mousp17556_c0_g1_i6	AT5G64290	dicarboxylate transport 2.1	2.11755201	0.00092571
Mousp16715_c1_g1_i6	AT2G33855	transmembrane protein	2.06354721	7.3282E-09
Mousp12292_c0_g1_i2	AT5G09650	pyrophosphorylase 6	2.02084136	1.0549E-06
Mousp15459_c0_g3_i1	AT1G44575	Chlorophyll A-B binding family protein	2.01519992	2.6424E-07
Mousp11032_c0_g1_i1	AT2G36930	zinc finger (C2H2 type) family protein	1.99550424	3.5079E-07
Mousp15459_c1_g1_i1	AT1G44575	Chlorophyll A-B binding family protein	1.96857574	1.5821E-06
Mousp11772_c0_g1_i3	AT1G22170	Phosphoglycerate mutase family protein	1.96630859	1.2869E-06
Mousp17393_c0_g3_i1	AT2G40490	Uroporphyrinogen decarboxylase	1.94764795	0.000013015
Mousp15227_c0_g1_i2	AT5G65230	myb domain protein 53	1.94157129	1.3559E-06
Mousp16477_c0_g4_i7	AT5G52975	egg cell-secreted-like protein (DUF1278)	1.92832108	0.00010985
Mousp15882_c0_g1_i1	AT2G19540	Transducin family protein / WD-40 repeat family protein	1.92449346	0.00096677
Mousp16664_c0_g3_i1	AT3G12410	Polynucleotidyl transferase, ribonuclease H-like	1.91165287	0.00028833
Mousp16466_c0_g1_i3	AT2G35120	Single hybrid motif superfamily protein	1.82700212	1.5271E-06
Mousp15769_c0_g1_i1	AT4G24230	acyl-CoA-binding domain 3	1.8206261	0.00013362
Mousp16717_c0_g1_i10	AT3G19430	late embryogenesis abundant	1.82053718	0.00022751
Mousp17443_c0_g1_i9	AT4G35000	ascorbate peroxidase 3	1.81353441	2.5071E-07
Mousp12426_c0_g1_i4	AT5G22140	FAD/NAD(P)-binding oxidoreductase family protein	1.79593008	0.00026667
Mousp15265_c0_g1_i8	AT5G02160	transmembrane protein	1.73674051	0.00003623
Mousp14642_c0_g1_i3	AT4G15520	tRNA/rRNA methyltransferase (SpoU) family protein	1.73401135	0.00087276
Mousp12053_c0_g1_i1	AT5G49300	GATA transcription factor 16	1.70744995	0.000012895
Mousp17024_c0_g1_i31	AT1G29900	carbamoyl phosphate synthetase B	1.69430823	0.00029494
Mousp14546_c0_g1_i2	AT5G48300	ADP glucose pyrophosphorylase 1	1.67256288	0.000055648
Mousp16932_c4_g2_i3	AT1G78430	ROP interactive partner 2	1.67009459	0.00026667
Mousp17693_c0_g2_i9	AT5G04270	DHHC-type zinc finger family protein	1.6547086	0.000030487
Mousp15496_c2_g7_i4	AT1G20140	SKP1-like 4	1.63691483	0.00012795
Mousp14376_c0_g1_i13	AT3G21150	B-box 32	1.62615568	0.00022751
Mousp16045_c0_g1_i2	AT5G13680	IKI3 family protein	1.61640969	0.000031256
Mousp17530_c2_g2_i38	AT3G63380	ATPase E1-E2 / haloacid dehalogenase-like hydrolase	1.61216412	0.00086341
Mousp13515_c0_g1_i2	AT5G12180	calcium-dependent protein kinase 17	1.60553028	0.000012596
Mousp17689_c0_g1_i7	AT4G30990	ARM repeat superfamily protein	1.59876228	0.00092316
Mousp10275_c0_g1_i1	AT4G14890	2Fe-2S ferredoxin-like superfamily protein	1.58967395	0.0003602
Mousp17110_c0_g2_i14	AT3G05060	NOP56-like pre RNA processing ribonucleoprotein	1.51185847	0.00051743
Mousp16289_c1_g3_i6	AT3G45190	SIT4 phosphatase-associated family protein	1.51073535	0.00052452
Mousp13265_c0_g1_i2	AT1G13580	LAG1 longevity assurance-like protein	1.47973166	0.000049111
Mousp17103_c0_g1_i2	AT5G48630	Cyclin family protein	1.45228723	0.00043854
Mousp15997_c0_g1_i6	AT5G37850	pfkB-like carbohydrate kinase family protein	1.45112666	0.00029494
Mousp15166_c1_g3_i2	AT4G27600	pfkB-like carbohydrate kinase family protein	1.44542141	0.00037996
Mousp17238_c0_g3_i3	AT3G43520	Transmembrane proteins 14C	1.36066059	0.00067169
Mousp16518_c0_g3_i2	AT4G23890	NAD(P)H-quinone oxidoreductase subunit S	1.35071287	0.00040989
Mousp17358_c1_g1_i9	AT3G04460	peroxin-12	1.3027287	0.00081849
Mousp17756_c0_g1_i13	AT4G22890	PGR5-LIKE A	1.30095184	0.0007896
Mousp13036_c1_g1_i1	AT4G29350	profilin 2	1.2830376	0.00092482
Mousp17102_c1_g1_i1	AT3G61070	peroxin 11E	1.26175474	0.00093898

Differential changes in transcript abundance (*FC*, fold change) in the samples taken 4h after submergence in liquid medium were calculated versus solid control and log_2_-transformed using edgeR; *FDR* false discovery rate denotes Benjamini-Hochberg-corrected *p* values; *A.t. Arabidopsis thaliana*

A *Mougeotia* sp. transcript homologous to *AtABA3* corresponded to the highest gene expression change (i.e. differential change in transcript abundance); it was up-regulated 154.9-fold following the shift from dry to wet. *ABA3* codes for a cytosolic molybdenum cofactor sulfurase that converts the carotenoid-derived abscisic aldehyde into the phytohormone abscisic acid (ABA). Despite the fact that several Zygnematophyceae have genes for the ABA receptors (de Vries et al. 2018; Cheng et al. 2019), these likely act in an ABA-independent function (Sun et al. 2019). We interpret the induction of the *ABA3* homolog rather as a readout of the aforementioned regulation of pigments (in this case, carotenoid metabolism) and photosynthesis-associated genes expression patterns that impact overall plastid physiology. In line with this, we also found regulation of violaxanthin de-epoxidase (4.8-fold upregulation) and a carotenoid cleavage dioxygenase (a homolog of CCD1; 10.6-fold upregulation). Carotenoid cleavage-derived metabolites are well known signaling molecules in plant cells—especially elicited upon environmental cues (Hou et al. 2016). Indeed, heat-induced changes in the expression of CCDs were observed for *Mougeotia* sp. (de Vries et al. 2020). Another aspect that needs to be taken into consideration is the adjustment of pigment profiles upon acclimating to a changing habitat; in an aquatic environment, not only the intensity but also the quality of light differs. Here, *Mougeotia* is a system rich in experimental history: in this algal genus, extensive work on chloroplast movement dependent on light qualities sensed by photoreceptors were carried out (Wagner and Klein 1981). Interestingly, Zygnematophyceae such as *Mougeotia* stand out by having chimeric photoreceptors containing domains of the red light phytochromes and blue light phototropins, the so-called neochromes (in our assembly *Mousp17450_c0_g1*; Data S1; (Suetsuga et al. 2006; Li et al. 2015). Responses regulated by these photoreceptors include chloroplast movement (note some of the twisting chloroplasts in Figure 3). We did not find clear signs for the differential regulation of genes related to light quality signaling (e.g. non-significant 2-fold downregulation of the phytochrome B homolog *Mousp17540_c0_g1*); further, the neochrome transcript *Mousp17450_c0_g1* was induced upon submergence, with an average TPM of 0.15 in solid control and 0.55 upon 4h submergence—however, as the numbers give away, it was expressed at such a low level that it was excluded from the analyses (see Material and Methods). Overall, it is conceivable that sensing the different spectral qualities of light when shifting to submergence is important and deserves further investigation.

**Fig. 5:**
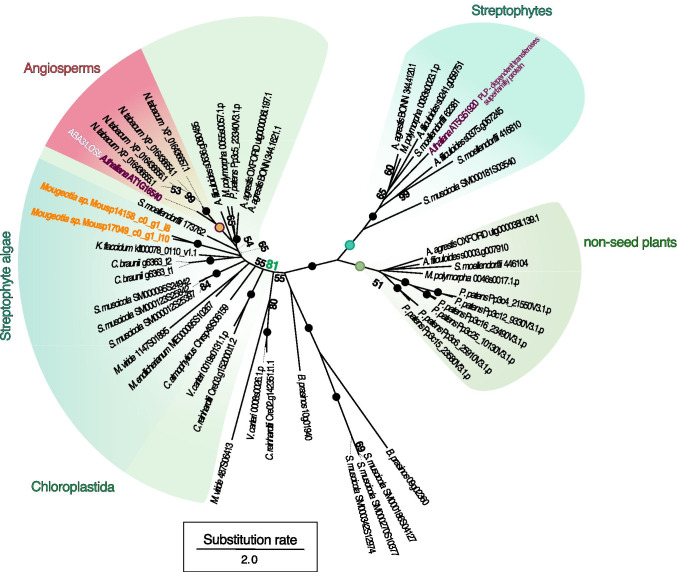
Phylogenetic framework for the putative ABA3 sequences identified in *Mougeotia* sp. MZCH 240. Phylogeny of homologs for the molybdenum cofactor sulfurase ABA3. Two homologs of ABA3 (Mousp14158_c0_g1_i8, Mousp17049_c0_g1_i10), the first of which was the most up-regulated gene in *Mougeotia* sp. MZCH 240 upon submergence, were aligned with 48 ABA3 homologs detected in diverse land plants, streptophyte algae, and chlorophyte algae. Homologs were aligned and an unrooted maximum-likelihood phylogeny was computed using WAG+F+I+G4 (chosen according to BIC) as model for protein evolution and 100 bootstrap replicates. Bootstrap values <50 are not shown in the figure; maximum bootstrap support is indicated by a filled dot

To explore whether the *Mougeotia* sp. *ABA3* homolog we detected is likely an *ABA3* ortholog, we performed a phylogenetic analysis. We used BLASTp to mine a phylodiverse protein dataset for ABA3 homologs, MAFFT (Katoh and Standley 2013) to align all putative ABA3 sequences, and IQ-TREE (v1.5.5; Nguyen et al. 2015) to construct a maximum likelihood phylogeny (Figure 5). The putative ABA3 homolog detected in *Mougeotia* sp. (Mousp14158_c0_g1_i8) fell, together with a potential paralog (Mousp17049_c0_g1_i10), into a moderately supported (65% bootstrap value) clade of land plant sequences. This clade was, however, nested in a more highly supported (81% bootstrap) clade of putative molybdenum cofactor sulfurases from across Chloroplastida. Thus, the ABA3 homolog detected in *Mougeotia* sp. seems to fall into the orthogroup of ABA3-type Molybdenum cofactor sulfurases that is conserved across Chloroplastida.

Green algae and land plants store photosynthate as starch. The buildup of starch appears to depend on the action of PLANT GLYCOGENIN-LIKE STARCH INITIATION PROTEINs (PGSIP; Chatterjee et al. 2005). Interestingly, we found a homolog of *PGSIP5* (AT1G08990) that is strongly induced (28.0-fold up) upon submergence. In light of the changes to the photosynthesis machinery, it is logical to also find genes associated with the downstream buildup of water-insoluble starch; the buildup of reserves appears a common theme among filamentous Zygnematophyceae that are challenged with environmental fluctuations (Pichrtová et al. 2016; Arc et al. 2020; de Vries and Ischebeck 2020). Indeed, the only enriched GO-term process was among the downregulated genes; there, we found that the GO-term “cellular carbohydrate catabolic process” (GO:0044275; *p* value 6.71x10-4) was enriched.

A homolog of a gene encoding a purple acid phosphatase (PAP) was found as the second most downregulated *Mougeotia* sp. gene (Mousp11308_c0_g1_i1; 301.0-fold downregulated); the resulting *Mougeotia* sp. protein bears a signal peptide (likelihood of 0.99 on TargetP-2.0), thus resembling the repertoire of secreted land plant PAPs with diverse functions in response to shifts in environmental conditions and nutrient availability (Bozzo et al. 2002; Kaida et al. 2010; Wang et al. 2011). It is noteworthy that, in a phylogenetic analysis, the *Mougeotia* sp. PAP fell into a clade of chlorophyte and streptophyte green algae, which formed a monophylum distinct from land plant PAPs (Figure S3).

Finally, we found differentially expressed *Mougeotia* sp. genes that are classically associated with pathogen response, including a gene putatively encoding a leucine-rich repeat transmembrane protein, (homologs of AT3G14840 and AT4G20140 were 91.0 and 7.7-fold up, respectively) and CAP (Cysteine-rich secretory proteins, Antigen 5, and Pathogenesis-related 1 protein; AT5G26130; 27.1-fold up). Such proteins are, however, equally often a sign of stress elicited by various changes in the environment (Creff et al. 2019 (AT4G20140); Le et al. 2014 (AT3G14840), Chien et al. 2015 (AT5G26130))—they might simply be a readout of the interwoven network that underpins environmental sensing. In line with this, a gene homologous to protein kinase-encoding *AT5G02290* showed clear induction (48.6-fold upregulation); this kinase might be involved in various signaling processes and speaks to the response of *Mougeotia* sp. to the changing environment. Indeed, several genes that speak to a general stress response were up-regulated. These included five LATE EMBRYOGENESIS ABUNDANT (LEA) homologs (6.3-fold, 5.2-fold, 5.1-fold, 4.7-fold, and 3.5-fold up-regulated), which are classical factors responsive to various abiotic stressors in other systems (Ingram and Bartels 1996; Hundertmark and Hincha 2008).

## Conclusion

We observed that submergence of *Mougeotia* triggered a conspicuous set of differentially regulated genes associated with changes in several photosynthesis and primary carbon metabolic pathways, suggesting remodeling of the photosystem apparatuses. This notion is supported by the observation that (a) various other photosynthesis-associated genes changed their expression and (b) slight but significant changes in the photochemical performance measured through the maximum quantum yield (*F*_v_/*F*_m_) were observed. Additionally, genes that speak to a remodeling of the pigment composition were regulated. It is conceivable that the composition of accessory pigments is being adjusted in response to the altered quality of light triggered by submergence. Altogether, our data suggest that some of the foremost adjustments that these filamentous zygnematophyceaen algae undergo during dry-to-wet transition are related to photophysiological acclimation; an assessment of the degree to which this holds true in the ecophysiological setting of temporary freshwater bodies is bound to be illuminating.

## Supplementary Information

Supplementary Fig. S1 Maximum quantum yield of photosystem II (PSII) (Fv/Fm) in Mougeotia scalaris SAG 164.80. A First and B second experimental setup of Fv/Fm measurements in solid and liquid control samples as well as samples exposed to the shift from agar to liquid (submergence). Before treatment, samples were grown for 7 days on WHM-Medium at 20°C, 120 μmol quanta m-2 s-1 in 9 cm petri dishes. “Liquid shift” samples (submergence) were treated by adding 10 ml liquid WHM-medium onto the agar surface and incubated up to 4h (A) or up to 24h (B) Fv/Fm was measured using an ImagMAX/L with an IMAG-K5 CCD camera. Details on the measurement settings are listed in the “Material and methods” section. Solid control samples are depicted in grey, liquid control samples are shown in blue, liquid treated samples (submergence) are depicted in pink. Statistical analysis was done using Mann–Whitney U tests, using R (version 3.6.1); significant differences are depicted using letters with p < 0.05. (PNG 96 kb)

High Resolution (EPS 282 kb)

Supplementary Fig. S2 All up-/downregulated genes in Mougeotia sp. MZCH 240 based on the log FC with the corresponding log TPM values (transcripts per million). All upregulated genes a shown on the left heatmap in red, downregulated genes are shown on the right heatmap in blue. Only genes with a significant change in gene expression (Benjamini-Hochberg corrected p < 0.001) and with a differential gene expression (log2[fold changesubmergence/control], calculated using edgeR) of ≥ or ≤ 2-fold change in gene expression levels were considered. Using the R package pheatmap, the data was sorted and clustered. All in all, 120 genes were significantly upregulated after submergence in liquid medium while 171 genes were significantly downregulated. Highlighted in yellow are the genes also shown in the phylogenetic trees in Figures 4 and S3: the most upregulated gene (TRINITY_DN14158_c0_g1_i8) as well the second most downregulated gene (TRINITY_DN11308_c0_g1_i1). (PNG 3261 kb)

High Resolution (EPS 24531 kb)

Supplementary Fig. S3 Phylogenetic framework for Mougeotia sp. MZCH 240 Purple Acid Phosphatase (PAP). A homolog of PAP (Mousp11308_c0_g1_i1), which was second most downregulated gene in Mougeotia sp. MZCH 240 exposed to the shift to liquid medium was aligned with PAP homologs detected in diverse land plants, streptophyte algae, and chlorophyte algae (46 sequences in total) and a maximum likelihood phylogeny was constructed. Homologs were sampled from a dataset that contained predicted proteomes of representatives across the Chloroplastida lineage, aligned, and an unrooted maximum-likelihood phylogeny was computed using WAG+I+G4 for PAP (chosen according to BIC) as model for protein evolution and 100 bootstrap replicates. Bootstrap values <50 are not shown in the figure; maximum bootstrap support is indicated by a filled dot. (PNG 623 kb)

High Resolution (EPS 2283 kb)

Supplementary Fig. S4 Extended phylogenetic framework for the putative ABA3 sequences identified in Mougeotia sp. Phylogenetic analyses were performed as in main Figure 5, but with additional sequences from the transcriptome datasets of Zygnema circumcarinatum (de Vries et al. 2018) and Spirogyra pratensis (de Vries et al. 2020). (PNG 649 kb)

High Resolution (EPS 1248 kb)

Data S1 (FASTA 6 kb)

## Data Availability

All data generated or analyzed during this study are included in this published article (and its supplementary information files), and the public databases of the NCBI: all RNAseq read data have been uploaded to the NCBI SRA. The reads from the control samples are available under the run IDs SRR9083693, SRR9083694, SRR9083695, SRR9083697, SRR9083698, SRR9083699; liquid treatment is available under the run IDs SRR9083681, SRR9083682, SRR9083688 (https://www.ncbi.nlm.nih.gov/sra?term=SRP198800). The reference assembly is publicly available under NCBI BioProject PRJNA543475 (https://www.ncbi.nlm.nih.gov/bioproject/PRJNA543475).

## References

[CR1] Arc E, Pichrtová M, Kranner I, Holzinger A (2020). Pre-akinete formation in Zygnema sp. from polar habitats is associated with metabolite re-arrangement. J Exp Bot.

[CR2] Banks JA, Nishiyama T, Hasebe M (2011). The selaginella genome identifies genetic changes associated with the evolution of vascular plants. Science.

[CR3] Bolger AM, Lohse M, Usadel B (2014). Trimmomatic: a flexible trimmer for Illumina sequence data. Bioinformatics.

[CR4] Bowman JL, Kohchi T, Yamato KT (2017). Insights into Land Plant Evolution Garnered from the Marchantia polymorpha Genome. Cell.

[CR5] Bozzo GG, Raghothama KG, Plaxton WC (2002). Purification and characterization of two secreted purple acid phosphatase isozymes from phosphate-starved tomato (*Lycopersicon esculentum* ) cell cultures: secreted acid phosphatases of P_i_-starved tomato cells. Eur J Biochem.

[CR6] Chatterjee Manash, Berbezy P, Vyas D, Coates S, Barsby T (2005). Reduced expression of a protein homologous to glycogenin leads to reduction of starch content in Arabidopsis leaves. Plant Science.

[CR7] Cheng S, Xian W, Fu Y (2019). Genomes of subaerial Zygnematophyceae provide insights into land plant evolution. Cell.

[CR8] Chien PS, Nam HG, Chen YR (2015). A salt-regulated peptide derived from the CAP superfamily protein negatively regulates salt-stress tolerance in Arabidopsis. J Exp Bot.

[CR9] Christa G, Cruz S, Jahns P (2017). Photoprotection in a monophyletic branch of chlorophyte algae is independent of energy-dependent quenching (qE). New Phytol.

[CR10] Correa-Galvis V, Poschmann G, Melzer M (2016). PsbS interactions involved in the activation of energy dissipation in Arabidopsis. Nat Plants.

[CR11] Creff A, Brocard L, Joubès J, Taconnat L, Doll NM, Marsollier AC, Ingram G (2019). A stress-response-related inter-compartmental signalling pathway regulates embryonic cuticle integrity in Arabidopsis. PLoS Genet.

[CR12] de Vries J, Ischebeck T (2020). Ties between stress and lipid droplets pre-date seeds. Trends Plant Sci.

[CR13] de Vries PJR, Simons J, van Beem AP (1983). Sporopollenin in the spore wall of *Spirogyra* (Zygnemataceae, Chlorophyceae). Acta Botanica Neerlandica.

[CR14] de Vries J, de Vries S, Slamovits CH (2017). How embryophytic is the biosynthesis of phenylpropanoids and their derivatives in streptophyte algae?. Plant Cell Physiol.

[CR15] de Vries J, Curtis BA, Gould SB, Archibald JM (2018). Embryophyte stress signaling evolved in the algal progenitors of land plants. Proc Natl Acad Sci U S A.

[CR16] de Vries J, Vries S, Curtis BA (2020). Heat stress response in the closest algal relatives of land plants reveals conserved stress signaling circuits. Plant J.

[CR17] de Vries S, Fürst-Jansen JMR, Irisarri I, Dhabalia Ashok A, Ischebeck T, Feussner K, Abreu IN, Petersen M, Feussner I, de Vries J (2021). The evolution of the phenylpropanoid pathway entailed pronounced radiations and divergences of enzyme families. Plant J.

[CR18] Eden E, Navon R, Steinfeld I (2009). GOrilla: a tool for discovery and visualization of enriched GO terms in ranked gene lists. BMC Bioinform.

[CR19] FASTQC (2018) A quality control tool for high throughput sequence data. Available at www.bioinformatics.babraham.ac.uk/projects/fastqc. Accessed September 15, 2018.

[CR20] Foyer CH, Lelandais M, Kunert KJ (1994). Photooxidative stress in plants. Physiol Plant.

[CR21] Friedl T, Lorenz M (2012). The Culture Collection of Algae at Göttingen University (SAG): a biological resource for biotechnological and biodiversity research. Procedia Environ Sci.

[CR22] Fürst-Jansen JMR, de Vries S, de Vries J (2020). Evo-physio: on stress responses and the earliest land plants. J Exp Bot.

[CR23] Gerotto C, Morosinotto T (2013). Evolution of photoprotection mechanisms upon land colonization: evidence of PSBS-dependent NPQ in late Streptophyte algae. Physiol Plant.

[CR24] Guillard RRL, Smith WL, Chanley MH (1975). Culture of phytoplankton for feeding marine invertebrates. Culture of marine invertebrate animals.

[CR25] Haas BJ, Papanicolaou A, Yassour M (2013). De novo transcript sequence reconstruction from RNA-seq using the Trinity platform for reference generation and analysis. Nat Protoc.

[CR26] Herburger K, Holzinger A (2015). Localization and Quantification of Callose in the Streptophyte Green Algae Zygnema and Klebsormidium: Correlation with Desiccation Tolerance. Plant Cell Physiol.

[CR27] Holzinger A, Pichrtová M (2016). Abiotic stress tolerance of charophyte green algae: new challenges for omics techniques. Front Plant Sci.

[CR28] Holzinger A, Kaplan F, Blaas K, Zechmann B, Komsic-Buchmann K, Becker B (2014). Transcriptomics of desiccation tolerance in the streptophyte green alga *Klebsormidium* reveal a land plant-like defense reaction. PLoS One.

[CR29] Holzinger A, Albert A, Aigner S, Uhl J, Schmitt-Kopplin P, Trumhová K, Pichrtová M (2018). Arctic, antarctic, and temperate green algae Zygnema spp. under UV-B stress: vegetative cells perform better than pre-akinetes. Protoplasma.

[CR30] Hori K, Maruyama F, Fujisawa T (2014). Klebsormidium flaccidum genome reveals primary factors for plant terrestrial adaptation. Nat Commun.

[CR31] Hou X, Rivers J, León P (2016). Synthesis and function of apocarotenoid signals in plants. Trends Plant Sci.

[CR32] Hundertmark M, Hincha DK (2008). LEA (Late Embryogenesis Abundant) proteins and their encoding genes in Arabidopsis thaliana. BMC Genomics.

[CR33] Hutin C, Nussaume L, Moise N (2003). Early light-induced proteins protect Arabidopsis from photooxidative stress. Proc Natl Acad Sci U S A.

[CR34] Ingram J, Bartels D (1996). The molecular basis of dehydration tolerance in plants. Annu Rev Plant Physiol Plant Mol Biol.

[CR35] Irisarri I, Darienko T, Pröschold T, Fürst-Jansen JMR, Jamy M, de Vries J (2021). Unexpected cryptic species among streptophyte algae most distant to land plants. Proc R Soc B.

[CR36] Jahns P, Holzwarth AR (2012). The role of the xanthophyll cycle and of lutein in photoprotection of photosystem II. BBA-Bioenergetics.

[CR37] Jiao C, Sørensen I, Sun X (2020). The Penium margaritaceum Genome: Hallmarks of the Origins of Land Plants. Cell.

[CR38] Kaida R, Serada S, Norioka N (2010). Potential role for purple acid phosphatase in the dephosphorylation of wall proteins in tobacco cells. Plant Physiol.

[CR39] Kalyaanamoorthy S, Minh BQ, Wong TKF (2017). ModelFinder: fast model selection for accurate phylogenetic estimates. Nat Methods.

[CR40] Kanehisa M, Sato Y, Morishima K (2016). BlastKOALA and GhostKOALA: KEGG tools for functional characterization of genome and metagenome sequences. J Mol Biol.

[CR41] Karsten U, Lembcke S, Schumann R (2007). The effects of ultraviolet radiation on photosynthetic performance, growth and sunscreen compounds in aeroterrestrial biofilm algae isolated from building facades. Planta.

[CR42] Karsten U, Herburger K, Holzinger A (2014). Dehydration, temperature, and light tolerance in members of the aeroterrestrial green algal genus Interfilum (Streptophyta) from biogeographically different temperate soils. J Phycol.

[CR43] Katoh K, Standley DM (2013). MAFFT Multiple Sequence Alignment Software Version 7: improvements in performance and usability. Mol Biol Evol.

[CR44] Kitzing C, Karsten U (2015). Effects of UV radiation on optimum quantum yield and sunscreen contents in members of the genera *Interfilum, Klebsormidium, Hormidiella* and *Entransia* (Klebsormidiophyceae, Streptophyta). Eur J Phycol.

[CR45] Krause GH, Vernotte C, Briantais JM (1982). Photoinduced quenching of chlorophyll fluorescence in intact chloroplasts and algae. Resolution into two components. Biochim Biophys Acta.

[CR46] Lamesch P, Berardini TZ, Li D (2012). The Arabidopsis Information Resource (TAIR): Improved gene annotation and new tools. Nucleic Acids Res.

[CR47] Lang D, Ullrich KK, Murat F (2018). The Physcomitrella patenschromosome-scale assembly reveals moss genome structure and evolution. Plant J.

[CR48] Le MH, Cao Y, Zhang X-C, Stacey G (2014). LIK1, A CERK1-Interacting Kinase, Regulates Plant Immune Responses in Arabidopsis. PLoS ONE.

[CR49] Leebens-Mack JH, Barker MS, Carpenter EJ (2019). One thousand plant transcriptomes and the phylogenomics of green plants. Nature.

[CR50] Li B, Dewey CN (2011). RSEM: accurate transcript quantification from RNA-Seq data with or without a reference genome. BMC Bioinform.

[CR51] Li X-P, Björkman O, Shih C, Grossman AR, Rosenquist M, Jansson S, Niyogi KK (2000). A pigment-binding protein essential for regulation of photosynthetic light harvesting. Nature.

[CR52] Li F-W, Melkonian M, Rothfels CJ (2015). Phytochrome diversity in green plants and the origin of canonical plant phytochromes. Nat Commun.

[CR53] Li F-W, Nishiyama T, Waller M (2020). *Anthoceros* genomes illuminate the origin of land plants and the unique biology of hornworts. Nature Plants.

[CR54] Li F-W, Brouwer P, Carretero-Paulet L, Cheng S, de Vries J, Delaux P-M (2018). Fern genomes elucidate land plant evolution and cyanobacterial symbioses. Nature Plants.

[CR55] Mann HB, Whitney DR (1947) On a test of whether one of two random variables is stochastically larger than the other. Ann Math Stat:50–60

[CR56] Merchant SS, Prochnik SE, Vallon O (2007). The Chlamydomonas genome reveals the evolution of key animal and plant functions. Science.

[CR57] Mikhailyuk T, Glaser K, Holzinger A, Karsten U (2015). Biodiversity of Klebsormidium (Streptophyta) from alpine biological soil crusts (Alps, Tyrol, Austria, and Italy). J Phycol.

[CR58] Montané MH, Dreyer S, Triantaphylidès C, Kloppstech K (1997). Early light-inducible proteins during long-term acclimation of barley to photooxidative stress caused by light and cold: High level of accumulation by posttranscriptional regulation. Planta.

[CR59] Moreau H, Verhelst B, Couloux A (2012). Gene functionalities and genome structure in Bathycoccus prasinos reflect cellular specializations at the base of the green lineage. Genome Biol.

[CR60] Morris JL, Puttick MN, Clark JW (2018). The timescale of early land plant evolution. Proc Natl Acad Sci U S A.

[CR61] Müller P, Li XP, Niyogi KK (2001). Non-photochemical quenching. A response to excess light energy. Plant Physiol.

[CR62] Nguyen L-T, Schmidt HA, von Haeseler A, Minh BQ (2015). IQ-TREE: a fast and effective stochastic algorithm for estimating maximum-likelihood phylogenies. Mol Biol Evol.

[CR63] Nichols HW, Stein JR (1973). Growth media – freshwater. Handbook of Phycological Methods.

[CR64] Nishiyama T, Sakayama H, de Vries J (2018). The Chara Genome: secondary complexity and implications for plant terrestrialization. Cell.

[CR65] Ohama N, Sato H, Shinozaki K, Yamaguchi-Shinozaki K (2017). Transcriptional regulatory network of plant heat stress response. Trends Plant Sci.

[CR66] Peers G, Truong TB, Ostendorf E (2009). An ancient light-harvesting protein is critical for the regulation of algal photosynthesis. Nature.

[CR67] Permann C, Herburger K, Niedermeier M, Felhofer M, Gierlinger N, Holzinger A (2021). Cell wall characteristics during sexual reproduction of Mougeotia sp. (Zygnematophyceae) revealed by electron microscopy, glycan microarrays and RAMAN spectroscopy. Protoplasma.

[CR68] Pichrtová M, Remias D, Lewis LA, Holzinger A (2013). Changes in phenolic compounds and cellular ultrastructure of arctic and antarctic strains of Zygnema (Zygnematophyceae, Streptophyta) after exposure to experimentally enhanced UV to PAR ratio. Microb Ecol.

[CR69] Pichrtová M, Arc E, Stöggl W, Kranner I, Hájek T, Hackl H, Holzinger A (2016). Formation of lipid bodies and changes in fatty acid composition upon pre-akinete formation in Arctic and Antarctic Zygnema(Zygnematophyceae, Streptophyta) strains. FEMS Microbiol Ecol.

[CR70] Poulícková A, Zizka Z, Hasler P, Benada O (2007). Zygnematalean zygospores: morphological features and use in species identification. Folia Microbiol.

[CR71] Prochnik SE, Umen J, Nedelcu AM (2010). Genomic analysis of organismal complexity in the multicellular green alga *Volvox carteri*. Science.

[CR72] Regensdorff M, Deckena M, Stein M, Borchers A, Scherer G, Lammers M, Hänsch R, Zachgo S, Buschmann H (2018). Transient genetic transformation of Mougeotia scalaris (Zygnematophyceae) mediated by the endogenous α-tubulin1 promoter. J Phycol.

[CR73] Renault H, Werck-Reichhart D, Weng J-K (2019). Harnessing lignin evolution for biotechnological applications. Curr Opin Biotechnol.

[CR74] Rippin M, Becker B, Holzinger A (2017). Enhanced desiccation tolerance in mature cultures of the streptophytic green alga Zygnema circumcarinatum revealed by transcriptomics. Plant Cell Physiol.

[CR75] Rippin M, Pichrtová M, Arc E (2019). Metatranscriptomic and metabolite profiling reveals vertical heterogeneity within a Zygnemagreen algal mat from Svalbard (High Arctic). Environ Microbiol.

[CR76] Robinson MD, Oshlack A (2010). A scaling normalization method for differential expression analysis of RNA-seq data. Genome Biol.

[CR77] Robinson MD, McCarthy DJ, Smyth GK (2010). edgeR: A Bioconductor package for differential expression analysis of digital gene expression data. Bioinformatics.

[CR78] Schlösser UG (1994). SAG - Sammlung von Algenkulturen at the University of Göttingen Catalogue of Strains 1994. Botanica Acta.

[CR79] Sierro N, Battey JND, Ouadi S (2014). The tobacco genome sequence and its comparison with those of tomato and potato. Nat Commun.

[CR80] Suetsuga N, Mittmann F, Wagner G, Hughes J, Wada M (2006). A chimeric photoreceptor gene, *NEOCHROME*, has arisen twice during plant evolution. Proc Natl Acad Sci U S A.

[CR81] Sun Y, Harpazi B, Wijerathna-Yapa A (2019). A ligand-independent origin of abscisic acid perception. Proc Natl Acad Sci U S A.

[CR82] von Schwartzenberg K, Bornfleth S, Lindner A-C, Hanelt D (2013). The Microalgae and Zygnematophyceae Collection Hamburg (MZCH) — living cultures for research on rare streptophytic algae. Algol Stud.

[CR83] Wagner G, Klein K (1981). Mechanism of chloroplast movement in *Mougeotia*. Protoplasma.

[CR84] Wang L, Li Z, Qian W (2011). The arabidopsis purple acid phosphatase AtPAP10 is predominantly associated with the root surface and plays an important role in plant tolerance to phosphate limitation. Plant Physiol.

[CR85] Wang S, Li L, Li H (2020). Genomes of early-diverging streptophyte algae shed light on plant terrestrialization. Nature Plants.

[CR86] Wickett NJ, Mirarab S, Nguyen N (2014). Phylotranscriptomic analysis of the origin and early diversification of land plants. Proc Natl Acad Sci U S A.

[CR87] Wodniok S, Brinkmann H, Glöckner G (2011). Origin of land plants: do conjugating green algae hold the key?. BMC Evol Biol.

[CR88] Zimmer A, Lang D, Richardt S (2007). Dating the early evolution of plants: detection and molecular clock analyses of orthologs. Mol Gen Genomics.

