# Supplementary figures and images for: Submergence of the filamentous Zygnematophyceae Mougeotia induces differential gene expression patterns associated with core metabolism and photosynthesis
Source: Protoplasma. 2021 Dec 22;259(5):1157–74. doi: 10.1007/s00709-021-01730-1 (PMC9385824; doi:10.1007/s00709-021-01730-1)

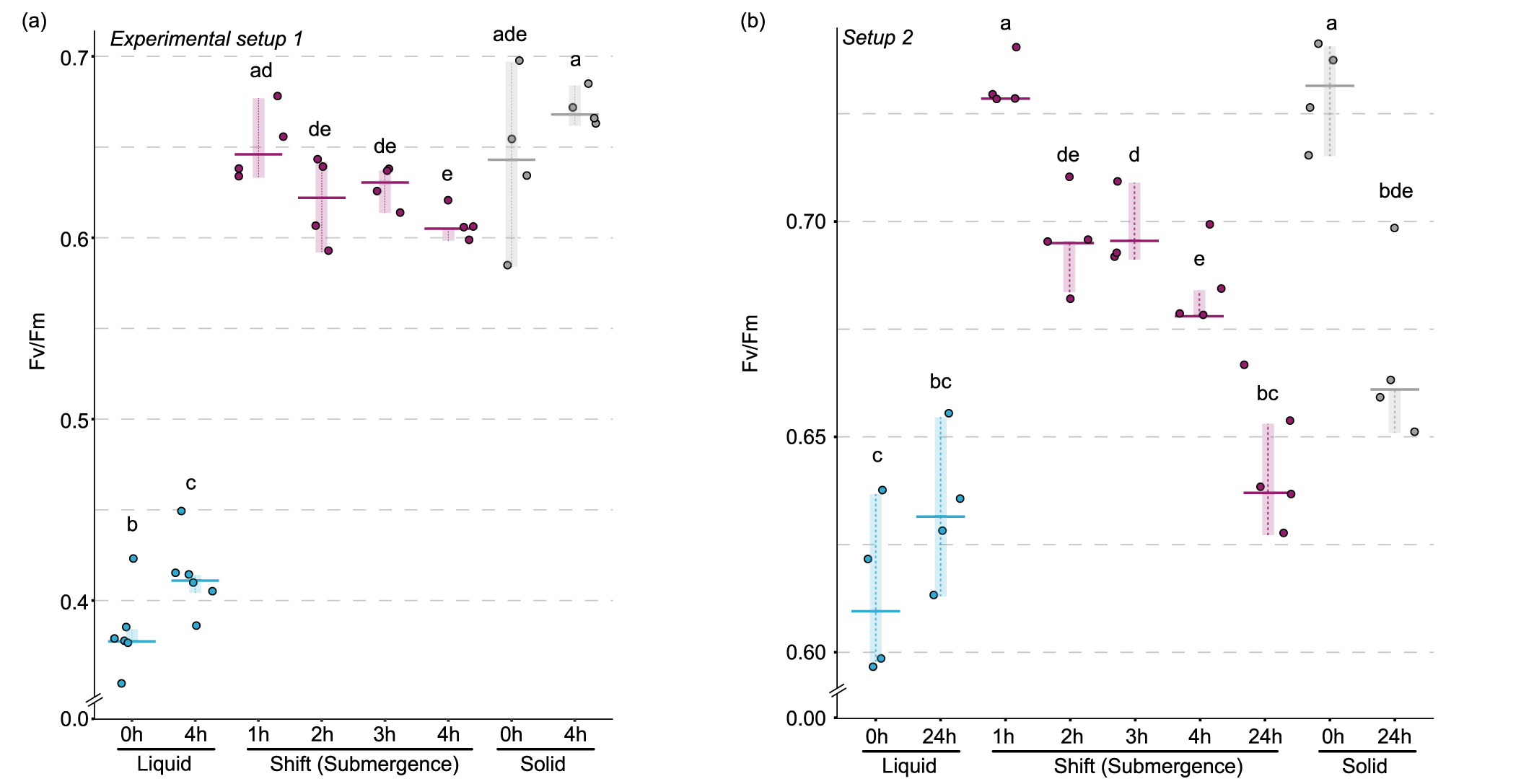

Supplement: Supplementary file 1 — Maximum quantum yield of photosystem II (PSII) (Fv/Fm) in Mougeotia scalaris SAG 164.80. A First and B second experimental setup of Fv/Fm measurements in solid and liquid control samples as well as samples exposed to the shift from agar to liquid (submergence). Before treatment, samples were grown for 7 days on WHM-Medium at 20°C, 120 μmol quanta m-2 s-1 in 9 cm petri dishes. “Liquid shift” samples (submergence) were treated by adding 10 ml liquid WHM-medium onto the agar surface and incubated up to 4h (A) or up to 24h (B) Fv/Fm was measured using an ImagMAX/L with an IMAG-K5 CCD camera. Details on the measurement settings are listed in the “Material and methods” section. Solid control samples are depicted in grey, liquid control samples are shown in blue, liquid treated samples (submergence) are depicted in pink. Statistical analysis was done using Mann–Whitney U tests, using R (version 3.6.1); significant differences are depicted using letters with p < 0.05. (PNG 96 kb) [file 709_2021_1730_Fig6_ESM.png]

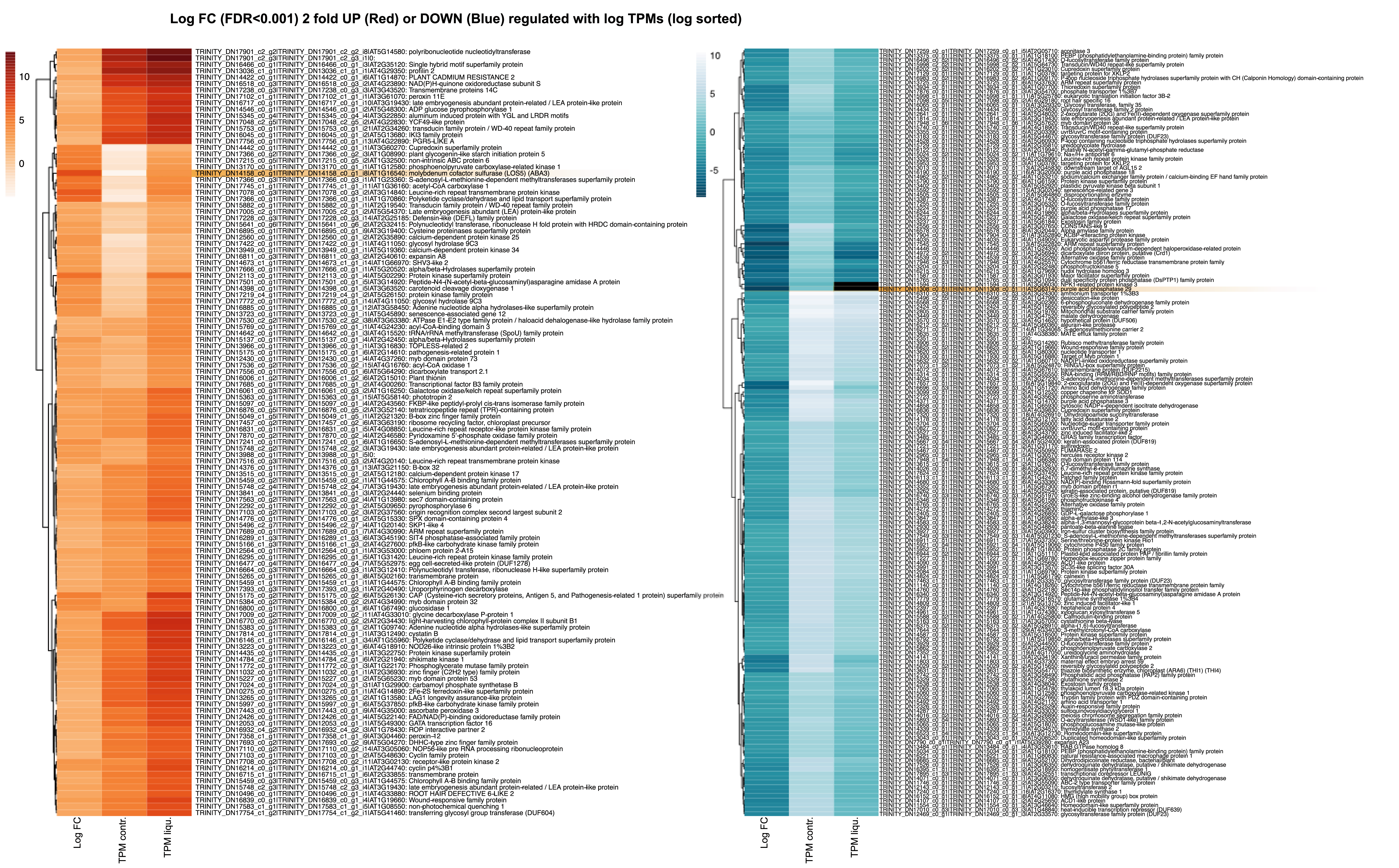

Supplement: Supplementary file 3 — All up-/downregulated genes in Mougeotia sp. MZCH 240 based on the log FC with the corresponding log TPM values (transcripts per million). All upregulated genes a shown on the left heatmap in red, downregulated genes are shown on the right heatmap in blue. Only genes with a significant change in gene expression (Benjamini-Hochberg corrected p < 0.001) and with a differential gene expression (log2[fold changesubmergence/control], calculated using edgeR) of ≥ or ≤ 2-fold change in gene expression levels were considered. Using the R package pheatmap, the data was sorted and clustered. All in all, 120 genes were significantly upregulated after submergence in liquid medium while 171 genes were significantly downregulated. Highlighted in yellow are the genes also shown in the phylogenetic trees in Figures 4 and S3: the most upregulated gene (TRINITY_DN14158_c0_g1_i8) as well the second most downregulated gene (TRINITY_DN11308_c0_g1_i1). (PNG 3261 kb) [file 709_2021_1730_Fig7_ESM.png]

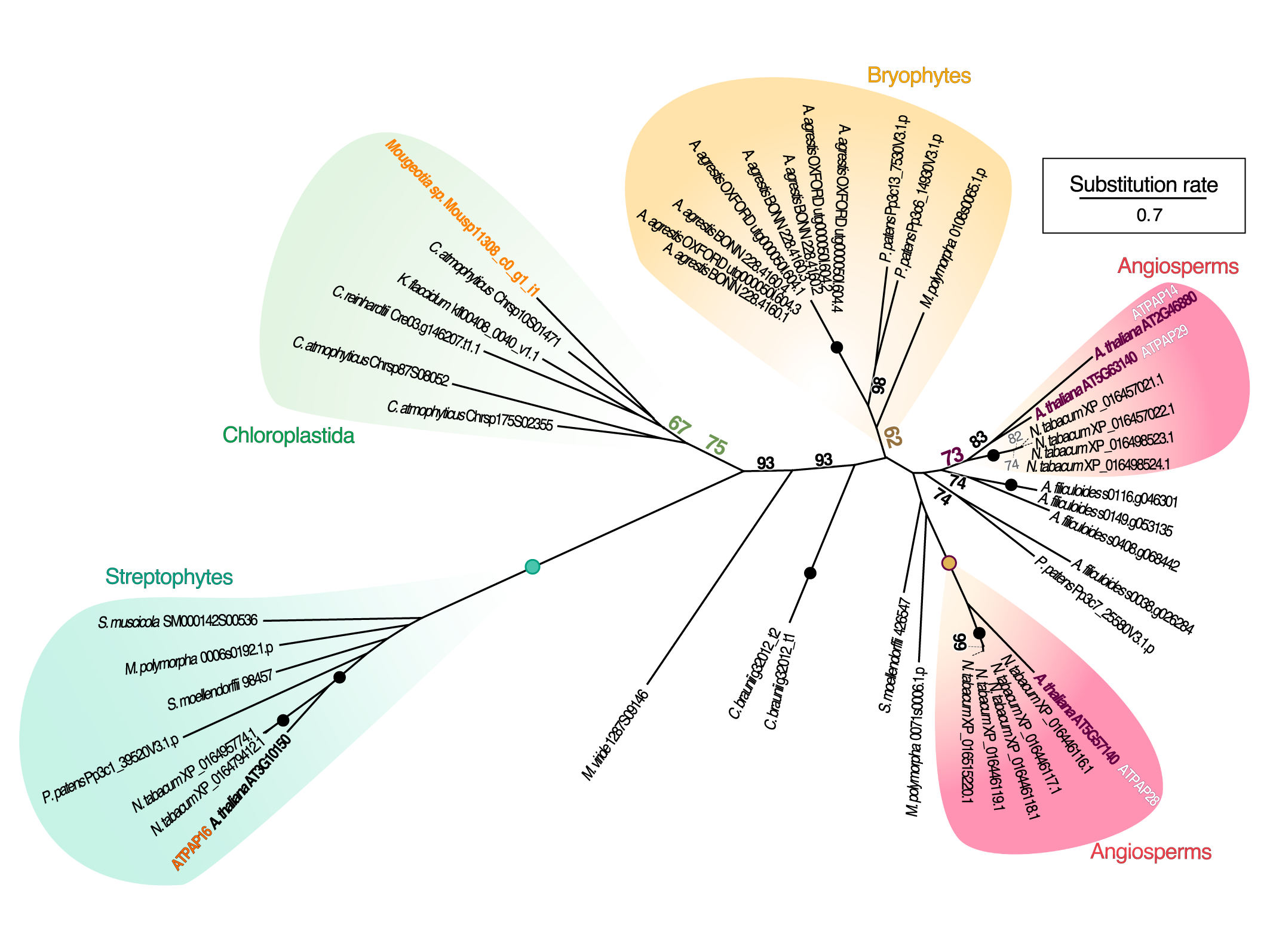

Supplement: Supplementary file 5 — Phylogenetic framework for Mougeotia sp. MZCH 240 Purple Acid Phosphatase (PAP). A homolog of PAP (Mousp11308_c0_g1_i1), which was second most downregulated gene in Mougeotia sp. MZCH 240 exposed to the shift to liquid medium was aligned with PAP homologs detected in diverse land plants, streptophyte algae, and chlorophyte algae (46 sequences in total) and a maximum likelihood phylogeny was constructed. Homologs were sampled from a dataset that contained predicted proteomes of representatives across the Chloroplastida lineage, aligned, and an unrooted maximum-likelihood phylogeny was computed using WAG+I+G4 for PAP (chosen according to BIC) as model for protein evolution and 100 bootstrap replicates. Bootstrap values <50 are not shown in the figure; maximum bootstrap support is indicated by a filled dot. (PNG 623 kb) [file 709_2021_1730_Fig8_ESM.png]

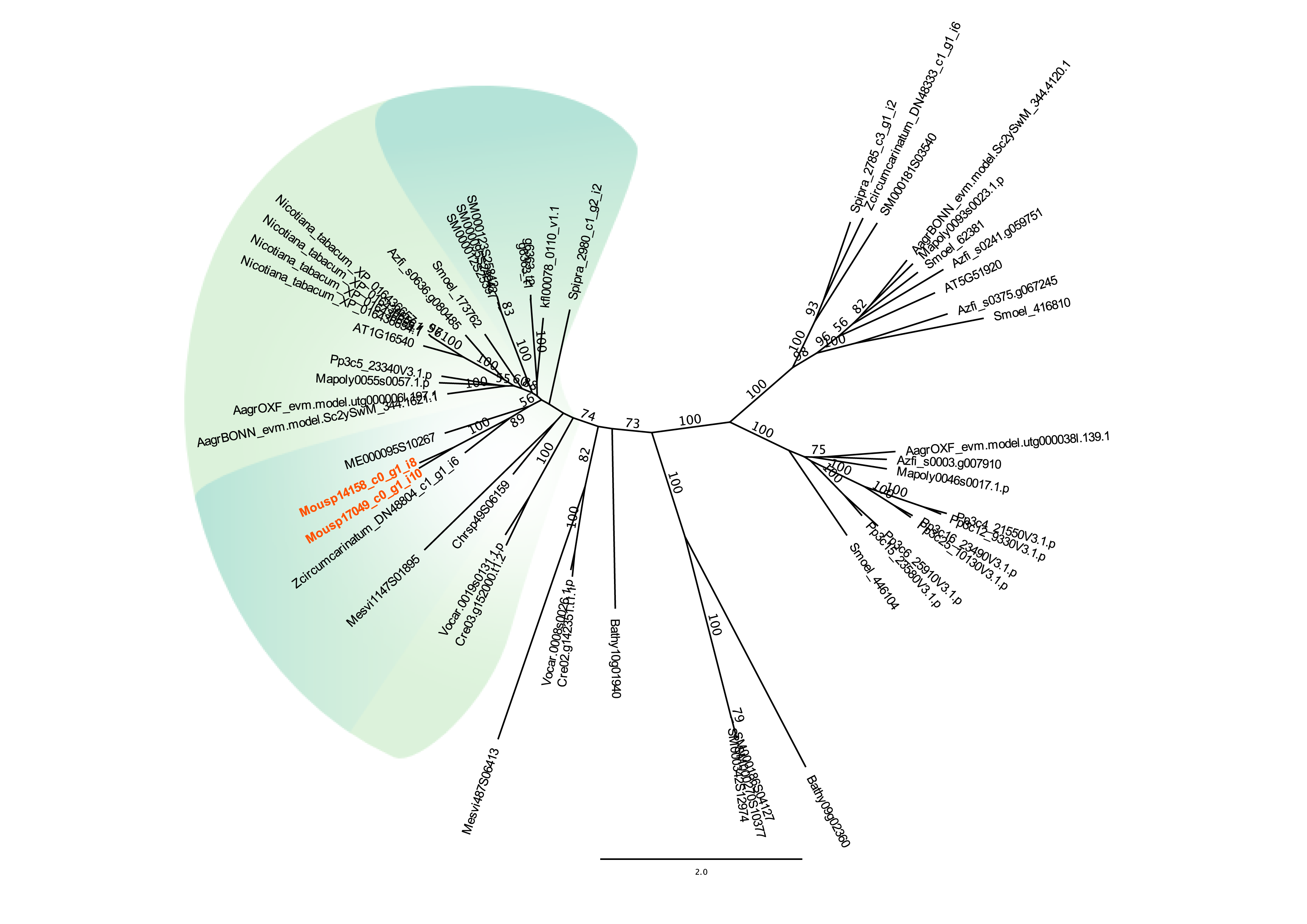

Supplement: Supplementary file 7 — Extended phylogenetic framework for the putative ABA3 sequences identified in Mougeotia sp. Phylogenetic analyses were performed as in main Figure 5, but with additional sequences from the transcriptome datasets of Zygnema circumcarinatum (de Vries et al. 2018) and Spirogyra pratensis (de Vries et al. 2020). (PNG 649 kb) [file 709_2021_1730_Fig9_ESM.png]
